# Transgenerational Actions of Environmental Compounds on Reproductive Disease and Identification of Epigenetic Biomarkers of Ancestral Exposures

**DOI:** 10.1371/journal.pone.0031901

**Published:** 2012-02-28

**Authors:** Mohan Manikkam, Carlos Guerrero-Bosagna, Rebecca Tracey, Md. M. Haque, Michael K. Skinner

**Affiliations:** Center for Reproductive Biology, School of Biological Sciences, Washington State University, Pullman, Washington, United States of America; Massachusetts General Hospital, United States of America

## Abstract

Environmental factors during fetal development can induce a permanent epigenetic change in the germ line (sperm) that then transmits epigenetic transgenerational inheritance of adult-onset disease in the absence of any subsequent exposure. The epigenetic transgenerational actions of various environmental compounds and relevant mixtures were investigated with the use of a pesticide mixture (permethrin and insect repellant DEET), a plastic mixture (bisphenol A and phthalates), dioxin (TCDD) and a hydrocarbon mixture (jet fuel, JP8). After transient exposure of F0 gestating female rats during the period of embryonic gonadal sex determination, the subsequent F1–F3 generations were obtained in the absence of any environmental exposure. The effects on the F1, F2 and F3 generations pubertal onset and gonadal function were assessed. The plastics, dioxin and jet fuel were found to promote early-onset female puberty transgenerationally (F3 generation). Spermatogenic cell apoptosis was affected transgenerationally. Ovarian primordial follicle pool size was significantly decreased with all treatments transgenerationally. Differential DNA methylation of the F3 generation sperm promoter epigenome was examined. Differential DNA methylation regions (DMR) were identified in the sperm of all exposure lineage males and found to be consistent within a specific exposure lineage, but different between the exposures. Several genomic features of the DMR, such as low density CpG content, were identified. Exposure-specific epigenetic biomarkers were identified that may allow for the assessment of ancestral environmental exposures associated with adult onset disease.

## Introduction

Epigenetic transgenerational inheritance provides an alternative molecular mechanism for germ line transmission of environmentally induced phenotypic change compared to that of classic genetics [1], [2]. Most factors do not have the ability to modify DNA sequence, but environmental factors such as nutrition or various toxicants can influence epigenetic processes to mediate alterations in genome activity [1], [3]. Environmental epigenetics focuses on how a cell or organism responds to environmental factors or insults to create altered phenotypes or disease. Previous observations have demonstrated that the exposure of a gestating female to the environmental fungicide compound vinclozolin [4] during fetal gonadal sex determination promotes a reprogramming of the male germ line epigenome [2]. The altered DNA methylation profile in the sperm becomes permanently reprogrammed to create an abnormal epigenome in the embryo and all cells and tissues derived from that embryo [5]. Later in life the animals develop adult onset disease states such as mammary tumors, prostate disease, kidney disease, testis abnormalities, and immune abnormalities at high (20–50%) frequencies [6]. Due to the imprinted-like nature of the altered epigenetic DNA methylation sites, the germ line (sperm) transmit this epigenome and adult onset disease phenotype to subsequent generations, which is termed epigenetic transgenerational inheritance [1]. The basic mechanism involves the ability of an environmental factor (compound) to alter the germ line DNA methylation program to promote imprinted-like sites that then transmit an altered epigenome that subsequently promotes adult onset disease phenotypes transgenerationally [1], [2]. The vast majority of environmental exposures act on somatic cells at critical windows of development to influence phenotype and/or disease in the individual exposed, but this will not become transgenerational [1], [3]. In the event the critical window for the primordial germ cell is affected by environmental exposure, the altered germ line has the ability to promote a transgenerational phenotype for subsequent generations [1]. More recently a number of reports have documented the ability of nutritional factors [7] and environmental toxicants such as bisphenol A (BPA), dioxin, vinclozolin and methoxychlor to promote epigenetic transgenerational inheritance [2], [8], [9], [10].

The current study was designed to investigate the potential epigenetic transgenerational actions of a variety of different toxicants or mixtures of relevant compounds. This was initiated to determine the compound specificity to promote epigenetic transgenerational inheritance and to determine if the epigenetic alterations may provide biomarkers for exposure. The environmental compounds (toxicants) selected have been shown to have biological and health effects [11], and were identified as common suspected exposures of military personnel. In addition, the cellular signal transduction process affected for each exposure is unique. The first compound mixture is termed “plastics” and includes bisphenol A (BPA) and the phthalates DEHP (bis(2-ethylhexyl)phthalate) and DBP (dibutyl phthalate) which are the combined exposures from most plastics that have been shown to promote *in vitro* and *in vivo* toxicologic effects [12]. Epigenetic effects of these compounds after neonatal exposures promotes adult onset disease [13], [14]. The second mixture involves the most commonly used human pesticide (permethrin) and insect repellent N,N-Diethyl-meta-toluamide (DEET), and is termed “pesticide” for this study, and has been shown to have some toxicologic effects in either *in vitro* or *in vivo* studies [15], [16], [17], [18], [19], [20]. The third compound used is dioxin (2,3,7,8-tetrachlorodibenzo-p-dioxin, TCDD), which has been shown to have significant *in vitro* and *in vivo* effects in the promotion of cellular abnormalities and adult onset disease states [21]. Epigenetic parameters have been shown to be influenced by dioxin actions [22]. The fourth exposure is jet fuel (jet propellant 8, JP8) which is a “hydrocarbon” mixture that is a significant environmental exposure due to its use for dust control on road surfaces [23]. Toxicological effects have been shown in *in vitro* and *in vivo* studies with JP8 exposures [24]. The four exposures used are common environmental toxicants which have been generally shown to promote abnormal or disease phenotypes. The objective of this study was to determine the potential ability for these different compounds and mixtures to promote epigenetic transgenerational inheritance of disease and map the potential alterations in the sperm epigenome.

The potential transgenerational diseases investigated focused on pubertal onset parameters and gonadal functions associated with infertility. The incidence of altered pubertal onset has increased over the past several decades in human populations [25], [26], [27]. Pubertal onset can occur several years early in some women [28]. This early onset of female puberty has been shown to affect brain development, endocrine organ systems and growth, that all potentially increase disease susceptibility later in life [29]. Although environmental exposures to endocrine disrupting compounds have been suggested as a causal factor [28], the basic mechanisms involved are unknown. The other disease parameters examined were associated with testis and ovary functions that influence fertility. In regards to testis function, sperm numbers and motility were examined, as well as spermatogenic cell apoptosis. In the human male population there has been a gradual decline in sperm number in most populations [30]. Estimates of male infertility appears to be over 10% in many human male populations [31]. In regards to ovarian function, the ovarian reserve or primordial follicle pool was assessed. An increasing percentage of the female population is developing premature ovarian failure associated with a loss of the follicle pool which promotes female infertility and affects approximately 15% of many female populations [32]. The causal factors for these gonadal disease phenotypes and increase in infertility are suggested to be due in part to environmental exposures to endocrine disruptor toxicants [33], but the basic molecular mechanisms involved are not known. The potential that the exposures used in the current study may promote the epigenetic transgenerational inheritance of these disease states is investigated.

## Results

The current study was designed to investigate the potential ability of various environmental compounds and mixtures to promote epigenetic transgenerational disease with a focus on pubertal and gonadal abnormalities. The alterations in the sperm epigenome were investigated to determine the similarities and differences between the different exposures on differential DNA methylation. The experimental design used pharmacologic doses, Table S1A, based on approximately 1% of the lethal oral dose 50% (LD50) for most of the compounds that previously have been shown *in vivo* to not cause direct toxicological effects. Gestating female outbred Harlan Sprague Dawley rats were given intraperitoneal (IP) injections daily between embryonic days 8–14 of fetal development correlating with gonadal sex determination. No consistent effects were observed on litter size, sex ratios or weaning weights, Figures S1 and S2. The number of litters and male and female animals obtained for each generation is presented in Table S1B and S1C. The F0 generation gestating female was the only animal injected IP. The F1 generation animals at 90 days of age were mated to the same lineage to generate the F2 generation and the F2 generation were mated to generate the F3 generation progeny as previously described [2]. No sibling or cousin breedings were used to avoid any inbreeding artifacts. No major overt toxicity was observed in F1, F2 or F3 generations, Figure S1 and S2. The only treatment that promotes some toxicity in the F1 generation was the high dose plastics, Table S1A, so a lower dose at 50% that shown in Table S1A was also used and termed “Low Dose Plastic” that had no toxicological effects, Figure S1. Anogenital distance was measured as an indicator of exposure to androgenic compounds that promote masculinization during the perinatal period [34], [35]. Analysis of anogenital index (AGI) demonstrated some effects of the treatments on the F2 and F3 generation animals, but no effects at the F1 generation animals, Figure S3. These actions on the AGI in the F2 generation are possibly due to the direct exposure of fetal germ cells to the endocrine disruptor activities of several of the exposure compounds (e.g. BPA, DEHP, DBP) [12], [13], [14], while the increased AGI in the F3 generation appears to be transgenerational. Therefore, classic endocrine disruptor actions [36] are likely not involved in the F2 and F3 generation, but only in the F1 generation. In considering the actions of environmental exposure the direct versus indirect (e.g. epigenetic) actions are critical. The exposure of the F0 generation gestating female directly affects the F0 generation female, the F1 generation embryo and the germ line inside the F1 embryo that will be generating the F2 generation animal [1]. Therefore, phenotypes in the F0, F1 and F2 generations may be due to direct exposures and are not transgenerational effects or phenotypes observed by definition. A transgenerational phenotype or phenomenon requires the lack of direct exposure to promote a generational effect [1], [3]. The actions on F0, F1 and F2 are due to a direct multi-generational exposure and only the F3 generation phenotype can be considered a transgenerational effect. Since the mechanisms promoting the F1 or F3 generation effects differ, the phenotypes can be distinct between the generations.

**Figure 1:**
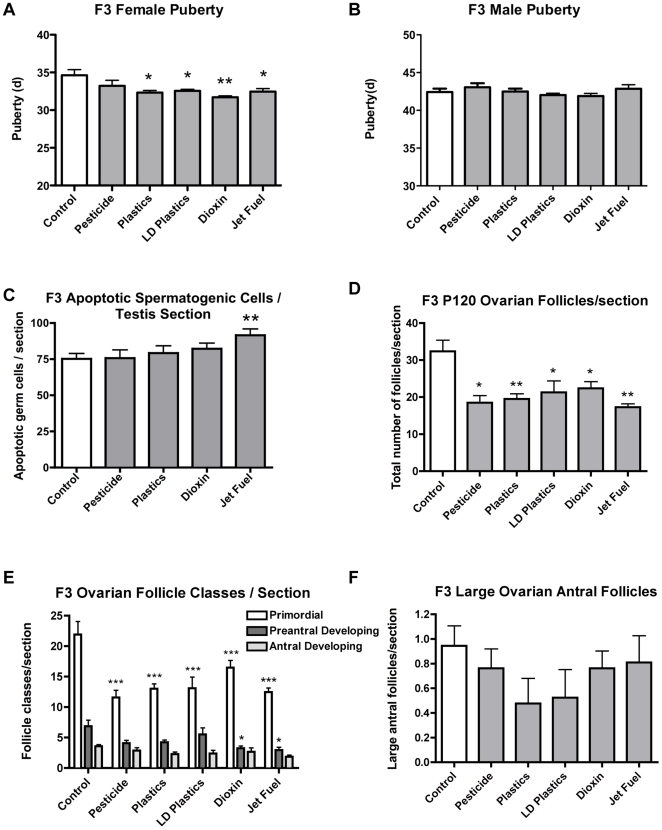
Ancestral (F0 generation female) exposures to environmental compounds promote transgenerational diseases, altering onset of puberty, testicular spermatogenic function and ovarian follicular development in F3 generation rat progeny. (A) Onset of female puberty was advanced from exposures to plastics, dioxin and jet fuel. (B) Onset of male puberty was unaffected from these exposures. (C) Increased apoptotic spermatogenic cells per testis section were observed from jet fuel exposure. (D) Total numbers of ovarian follicles per section were reduced in individuals from all exposures, (E) Total numbers of primordial follicles per section declined. (F) Total numbers of large ovarian antral follicles were unaffected. The animal n value is presented in Table S1C (*p<0.05; **p<0.01, ***p<0.001).

Puberty is a developmental process involving the hypothalamic – pituitary – gonadal axis which initiates during fetal development and matures in adolescence [25]. The onset of puberty was investigated with the different exposure lineages of control (DMSO vehicle), pesticide, low and high dose plastics, dioxin, or hydrocarbons in the F1–F3 generation rats. The analysis was initiated for females at postnatal day 30 and males at postnatal day 35 until puberty (vaginal opening or balano-preputial separation) [37]. In the F1 generation plastics promoted delayed female pubertal onset, while in the F2 generation plastics, dioxin and jet fuel promoted early onset of puberty for females, with plastics and dioxin promoting early onset of puberty in males, Figure S4. In the transgenerational F3 generation it was demonstrated that plastics, low dose plastics, dioxin and jet fuel promote early onset of puberty in females, while having no effect on males, Figure 1A, 1B. Therefore, several of the exposures were found to promote early onset of puberty in females transgenerationally.

Gonadal function for both testis and ovary were investigated in the F3 generation at postnatal 120 days of age. Previously vinclozolin was shown to promote a transgenerational phenotype of spermatogenic cell apoptosis [2], so potential germ cell apoptosis in the testis was investigated. The jet fuel exposure was found to transgenerationally increase spermatogenetic cell apoptosis in the F3 generation male testis, Figure 1C. Epididymal sperm concentration and motility for the F3 generation were also investigated and did not provide consistent alterations transgenerationally, as previously seen with vinclozolin exposure. The F3 generation ovaries were examined for total follicle number and the individual types of primordial follicles, primary follicles and developing follicles were categorized, Figure 1D and 1E. All the exposures were found to promote a transgenerational effect on the F3 generation ovary with a significant reduction in total follicle number, Figure 1D, and the follicle class primarily affected was the primordial follicle, Figure 1E. Therefore, all the exposures promoted the transgenerational phenotype of a reduction in the primordial follicle pool size. The large developing antral follicles were counted to determine potential effects on later stage follicle development and no differences were observed between the exposures when compared to control, Figure 1F. The transgenerational action of the various exposures on the ovary was a reduction in the primordial follicle pool size. This may promote premature ovarian failure as the animals age. The testis and ovary are hormone regulated and both produce endocrine steroids. Hormone levels were analyzed to determine how the endocrine system was responding transgenerationally. The F3 generation males had a reduction in testosterone levels in the plastics, dioxin and jet fuel exposure lineages, Figure S5A, while the females had no change in progesterone levels, Figure S5B. No change in luteinizing (LH) hormone levels was detected in either male or female F3 generation animals, Figure S5C & D. Therefore, the endocrine system was altered transgenerationally in the males.

**Figure 2:**
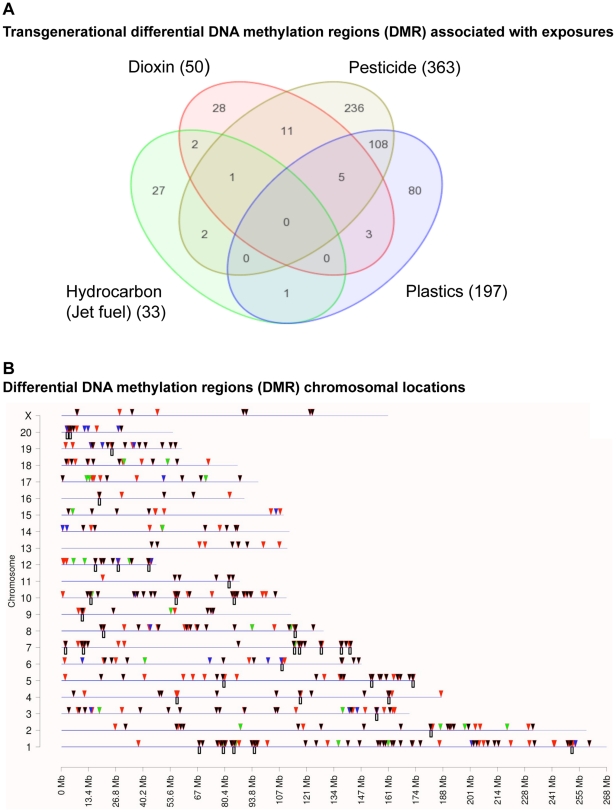
The transgenerational DMR associated with each exposure group identified. (A) Venn diagram of exposure DMR lists of F3 generation rat genes with differential DNA methylation due to *in vivo* exposure of F0-generation gestating female with Dioxin, Pesticide, Plastics or Hydrocarbons/Jet fuel. (B) Chromosomal location of each exposure group DMR are indicated with red arrow (plastics), green arrow (dioxin), blue arrow (hydrocarbon) and black arrow (pesticide). The chromosome number and size are indicated. The box below the line indicates DMR cluster in 2–5 megabase regions with statistical significance (p<0.05).

**Figure 3:**
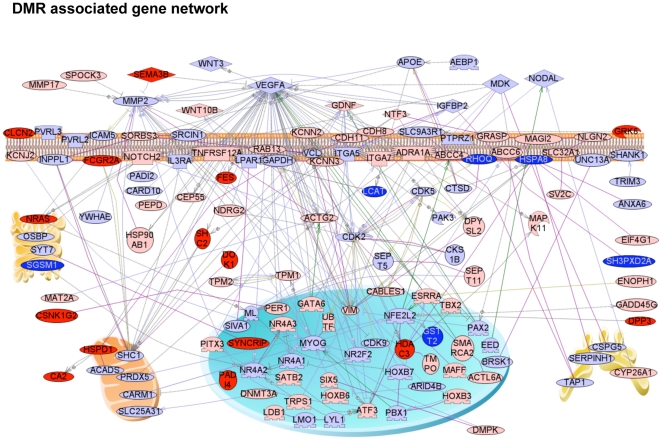
Direct connection gene sub-network for combined genes with transgenerational DMR associated exposures for Dioxin (red shapes), Pesticide (light blue shapes), Plastics (pink shapes) or Hydrocarbons/Jet fuel (dark blue shapes) indicated. Only 140 directly connected genes out of 499 unique genes associated with the combined lists are shown. Node shapes code: oval and circle – protein; diamond – ligand; circle/oval on tripod platform – transcription factor; ice cream cone – receptor; crescent – kinase or protein kinase; irregular polygon – phosphatase. Arrows with plus sign show positive regulation/activation, arrows with minus sign – negative regulation/inhibition; grey arrows represent regulation, lilac – expression, purple – binding, green – promoter binding, and yellow/olive – protein modification.

The mechanism involved in these transgenerational phenotypes is the reprogramming of the germ line (sperm) during male sex determination [1], [3]. This altered sperm epigenome appears to be permanently reprogrammed and escapes the DNA methylation programming at fertilization to allow transgenerational transmission of the altered sperm epigenome, that then promotes all tissues developed from that sperm to have altered cell and tissue transcriptomes that can promote transgenerational disease [1]. Previously, vinclozolin was shown to promote a transgenerational (F3 generation) alteration in DNA methylation [2], [5] and a transgenerational transcriptome alteration in tissues like the testis [38]. The F3 generation rat sperm from the control and all exposure groups were collected for genome wide promoter DNA methylation analysis [5]. The procedure involved the use of an antibody to methylcytosine to immunoprecipitate methylated DNA (MeDIP) and then a competitive hybridization tiling array (Chip) for a MeDIP-Chip analysis [5]. Differentially methylated sites were identified for all the different exposure lineages in the F3 generation sperm when compared with vehicle control F3 generation sperm. The complete lists of differential methylation regions (DMR) for each exposure in the F3 generation sperm are provided in Table S2(A–D). The overlap of the DMR sets for each exposure is shown in a Venn diagram in Figure 2A. The number of DMR for hydrocarbons (jet fuel) was 33, dioxin 50, plastics (BPA, DEHP, DBP) 197 and pesticide (permethrin and DEET) 363 with a statistically significant difference (p<10^−5^). Interestingly, the majority of each DMR set was specific to an exposure group and not common with the other exposure DMRs. The only exception was an overlap between plastics and pesticide of 113 DMRs, Figure 2A. Therefore, each exposure had a unique signature of epigenetic alterations in the F3 generation sperm. The chromosomal localizations of these sites are shown in Figure 2B. The DMRs are seen on all autosomes and the X chromosome. Clustering analysis of the DMRs when over represented in specific chromosomal locations identified 35 different clusters (2–5 megabase each) of DMR between the exposures that with z-score analysis have a statistically significant difference (p<0.05), Figure 2B. These DMR clusters may represent “epigenetic control regions” where different exposure DMRs may commonly regulate genome activity. The functional significance of these DMR clusters remains to be elucidated and are identified for individual DMR in Table S2. In considering the combined DMR and associated gene promoters for all exposures, the potential cellular signaling processes affected demonstrated similar pathways are predominant, as shown in Table S3. A gene network analysis for direct connections within the total gene set associated with the DMR is shown in Figure 3 and demonstrates extracellular, membrane, cytoplasmic and nuclear associated genes are all associated with the DMR identified. Common cellular signaling pathways and processes appear to be involved from the gene network identified. Therefore, common cellular pathways and gene networks may be influenced by the different exposures and transgenerational sperm epigenomes. Although exposure specific transgenerational differential DNA methylation regions (DMR) are predominant, the common cellular processes and gene networks effected may explain the similar disease phenotypes observed.

**Figure 4:**
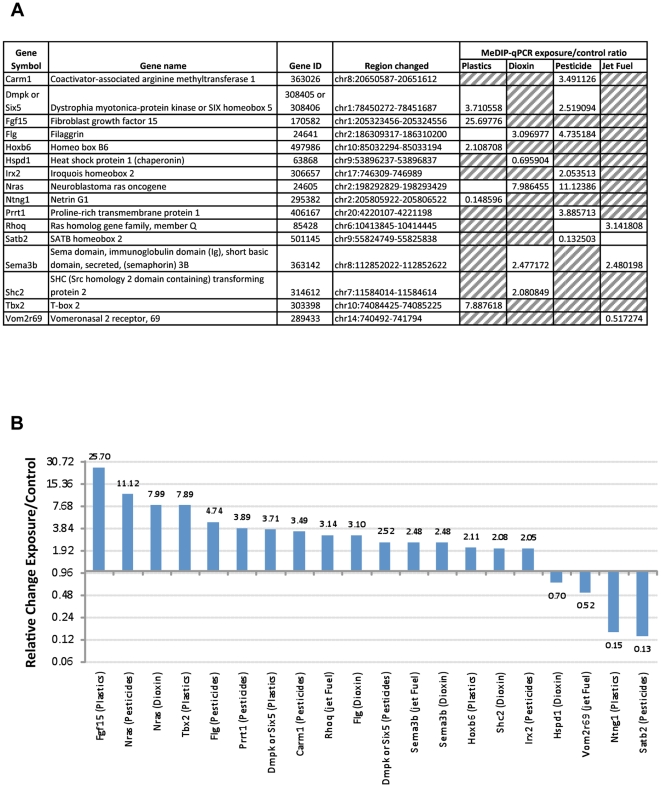
The MeDIP-qPCR analysis of (A) selected DMR for each exposure was used to confirm MeDIP-Chip analysis and (B) relative change (exposure/control) ratio presented for each DMR. All changes shown are statistically significant between control and exposure (p<0.05).

**Figure 5:**
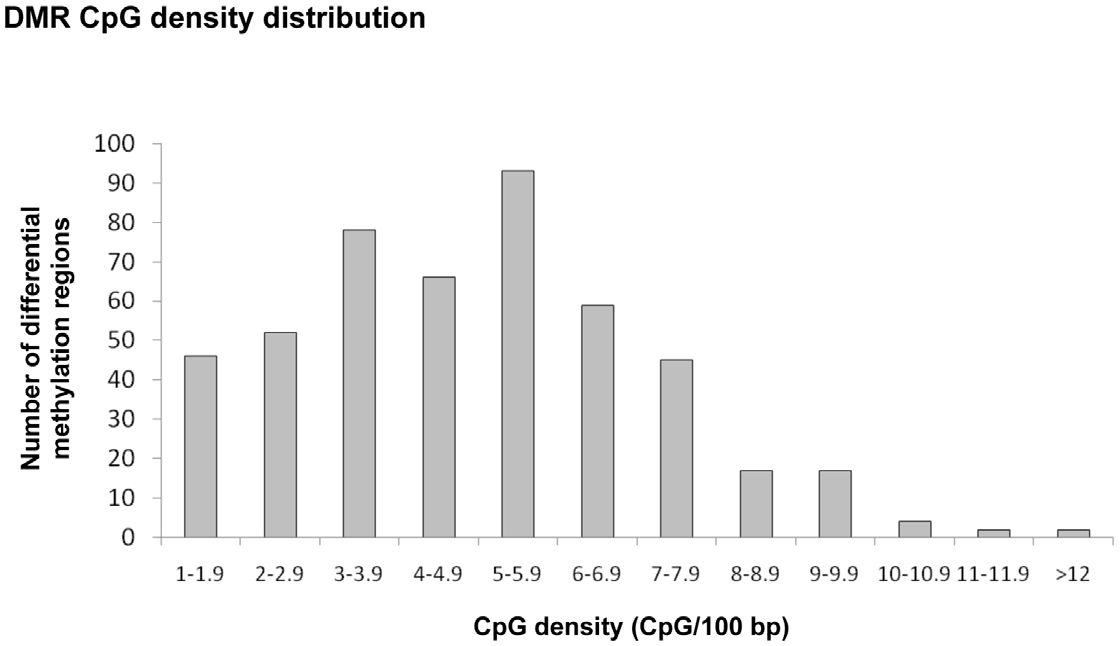
Differential DNA methylated region (DMR) CPG density distribution. The CpG density (CpG/100 bp) associated with all exposure DMR are presented with number of DMR on y axis and density (CpG per 100 bp) on x axis.

The identification of epigenetic alterations in specific regions of the F3 generation sperm support a role for epigenetic transgenerational inheritance of the disease phenotypes observed. Several of the top exposure specific DMRs for each exposure with the highest statistical significance were selected for confirmation with quantitative PCR of the MeDIP samples. A list of the confirmed exposure specific signatures are presented in Figure 4. In addition, several of the top overlapped (common) DMR were also selected and shown. The MeDIP qPCR analysis demonstrated both increases and decreases for the exposure specific and common DMR, Figure 4B. These exposure specific DMR are considered potential epigenetic biomarkers for exposure and the transmission of transgenerational phenotypes. Further analysis of the epigenetic sites identified considered two genomic features associated with the DMRs. The first one was a DNA sequence motif termed “Environmentally Induced DNA Methylation Region 1” (EDM1) that was previously identified and shown to be associated with a high percentage of the vinclozolin induced sperm DMRs [5]. This motif may not be at the specific altered DNA methylation site, but is within the 400–500 bp region. A DNA sequence motif such as EDM1 may promote a region of sensitivity for these DMR's to be programmed transgenerationally. The potential presence of this EDM1 motif in the epigenetic sites (DMR) identified in the current study for all the exposures was determined. An evaluation of the presence of EDM1 using the MCAST online software revealed a statistically significant higher EDM1 presence in promoter regions of the jet fuel and dioxin exposure groups (74.19% and 57.63%, respectively) compared to a computer generated random set of 144 promoters (20.83%). The presence of EDM1 in the promoter regions of the plastics (20.47%) and pesticides (7.36%) was similar or below its presence in the random set of promoters. This suggests that the molecular mechanisms involved in the targeting of these regions to produce a transgenerational change in DNA methylation may differ among the exposure groups. Another genomic feature investigated was the CpG density within the DMR identified. The frequency of CpG number per 100 bp for the DMR demonstrates the DMR identified for all exposures have an average CpG content of 4.9 CpG/100 bp with none above 15 CpG/100 bp, Figure 5. A small CpG cluster in a CpG desert appears to be a primary feature of the transgenerational DMR identified, and not shores or islands of CpG. Therefore, specific genomic features such as low CpG density, isolated CpG clusters, and the presence of a unique DNA sequence motif may be involved in facilitating the programming of these epigenetic sites (DMR) in the male germ line.

## Discussion

The current study used pharmacologic doses of all the compounds and mixtures based on approximately 1% of the oral LD50 dose for most exposures (compounds), Table S1A. The objective was to determine if these exposures have the capacity to promote epigenetic transgenerational inheritance of a disease phenotype, and not to do risk assessment of the exposures. Now that the current study has established the transgenerational actions of these compounds, risk assessment toxicological studies involving dose curves of relevant environmental doses are needed. The phenotypes observed may vary with the dose as shown with the plastics in the current study. Since the F1 generation involves direct exposure and the F3 generation is germ line mediated transgenerationally, the phenotypes can differ between the generations. In addition to considering the mode of administration and dose, the critical window of exposure to promote the epigenetic transgenerational phenotype is gonadal sex determination, which for the human is 6–18 weeks of gestation. The gestating women in the first half of pregnancy would be the population most sensitive to exposures of environmentally induced epigenetic transgenerational inheritance.

The transgenerational disease phenotype investigated focused on pubertal onset and gonadal function. It was previously observed with vinclozolin induced transgenerational adult onset rat disease [2], the majority of disease developed between 6–12 months of age [6]. Therefore, additional adult onset diseases are anticipated as the animals age, but remain to be investigated. In regards to pubertal onset the plastics, low dose plastics, dioxin and hydrocarbon (jet fuel) exposures promoted an early (precocious) pubertal onset, Figure 1, with no transgenerational effects on male pubertal onset, Figure S6. In the majority of developed countries early pubertal onset in girls has increased significantly in the past several decades [25], [28]. This precocious puberty can promote behavioral, mental and endocrine physiological effects in the female and increase the incidence of adult onset disease [28]. Previous studies have suggested environmental exposures of estrogenic endocrine disruptors may be in part the causal factor for this pubertal onset condition. The current study extends this hypothesis to not only consider the direct exposures of the female, but ancestral exposures of the previous generations. The potential that early pubertal onset may in part involve epigenetic transgenerational inheritance mechanisms now needs to be considered.

In considering gonadal function and fertility both the testis and ovary were investigated. The testis was found to have an increased spermatogenic cell apoptosis in the jet fuel hydrocarbon F3 lineage males, Figure 1. Previous observations with vinclozolin also showed a transgenerational spermatogenic cell apoptosis phenotype [2]. In many regions of the world human sperm numbers have declined [30] and male infertility has increased [31]. The potential that environmentally induced epigenetic transgenerational inheritance may be a factor in these disease conditions needs to be considered. In regards to ovarian function all the environmental exposures were found to promote a decline in total follicle numbers and specifically the primordial follicle pool size, Figure 1. The primordial follicle pool size is the ovarian reserve for oocyte (egg) production throughout reproductive life [39]. The primordial follicle pool develops early in fetal (human) or early postnatal (rodent) life and then can not increase, but declines with age. Human females enter menopause when the primordial follicle pool is exhausted. A premature loss of follicles promotes infertility and is termed premature ovarian failure (POF), which is associated in part with the dramatic increase in female infertility in many parts of the world [32]. Previously it was hypothesized that POF was primarily of genetic origin, but the current study suggests environmental exposures and epigenetic transgenerational inheritance may also be a significant factor in the disease etiology to increase female infertility and premature onset of menopause. The environmental induction of the pubertal, testis and ovarian transgenerational disease phenotypes suggests that environmental epigenetics and epigenetic transgenerational inheritance will be molecular factors to consider in these and other disease etiologies.

The environmental compounds and mixtures used in the current study are all reported to be major exposures for the general population and military personnel. The ability of epigenetics to be involved in the long term and transgenerational actions of these exposures needs to be further investigated. The current study documents the distinct actions of each exposure to promote a unique sperm epigenome alteration, Figure 2. Interestingly, these environmentally induced distinct epigenetic changes in differential DNA methylation regions (DMR) provide epigenetic biomarkers for ancestral environmental exposures. Each exposure had a distinct epigenetic signature that can be used as a biomarker. Although further research on individual animal variation, alterations in DMR in different cell types, and developmental effects on DMR are needed, the current study provides the proof of concept that epigenetic biomarkers for environmental exposures exist.

In addition to the identification of these ancestral epigenetic biomarkers in sperm, genomic features were identified that provide insight into why these sites may become permanently reprogrammed. A DNA sequence motif previously identified and termed “Environmentally Induced DNA Methylation Region 1 (EDM1)” [5] was found to be associated with a high percentage of the promoter regions of the hydrocarbon and dioxin exposure groups. Similar observations were previously made in examining the vinclozolin induced DMR in transgenerational sperm [5]. Interestingly, the plastics and pesticide exposure groups DMR did not have the presence of the EDM1 motif above background random promoter levels. Therefore, distinct molecular mechanisms may be involved in promoting the sensitivity of transgenerationally programmed DMR. This may include an alternate DNA sequence motif to be elucidated, or a more stochastic mechanism to be considered. The other genomic feature identified involved the CpG content or density associated with all the DMRs identified for all exposures. The previous dogma is that epigenetic modifications in CpG islands or shores with highest CpG density are critical. The DMRs identified had what is considered a low range CpG density [40] with an average of 8 CpG/100 bp content and no DMR with a CpG density greater than 15 CpG/100 bp, Figure 5. Therefore, the DMR appear to have small clusters of CpG in a CpG desert, as previously described [41]. Evolutionarily CpG deserts develop due to the high mutation rate of CpG sites. The maintenance of small CpG clusters in these deserts may suggest a conserved critical epigenetic regulatory site. These genomic features are speculated to have a role in how the DMR become permanently programmed and promote epigenetic transgenerational inheritance. The current study focuses on a genome wide analysis of promoters. Further investigation of genome wide effects and the role of these genomic features is now needed to provide further insights into the molecular elements of epigenetic transgenerational inheritance.

The transmission of epigenetic information between generations in the absence of any direct environmental exposures is defined as epigenetic transgenerational inheritance [1], [2], [3]. Therefore, in the case of exposure of a gestating female, only after the F3 generation can epigenetic transgenerational inheritance be considered [1]. The previous observations that vinclozolin and methyoxychlor induced epigenetic transgenerational inheritance [2] developed the question of compound specificity. The current study indicates different environmental compounds and mixtures with very different effects on signal transduction processes involved can all promote epigenetic transgenerational phenotypes. Therefore, the specific compound or signaling event does not appear critical, but instead any agent that can modify the normal development and differentiation of the primordial germ cell during gonadal sex determination [1], [3] can impact epigenetic programming and promote transgenerational inheritance. Although the majority of exposures will influence somatic cells and disease or phenotypes in the individual exposed, those actions that promote epigenetic transgenerational inheritance may have additional significant biological impacts. This includes providing a molecular mechanism for environmental toxicology, disease etiology, early life basis of adult onset disease [1], [3] and evolutionary biology [42]. The availability of ancestral environmental epigenetic biomarkers is anticipated to significantly facilitate the research in these areas of science.

## Materials and Methods

### Animal studies

All experimental protocols for the procedures with rats were pre-approved by the Washington State University Animal Care and Use Committee (IACUC approval # 02568-026). The University Department of Environmental Health and Safety approved all the protocols for the use of hazardous chemicals in this experiment. Sprague Dawley SD female and male rats of an outbred strain (Harlan) at about 70 and 100 days of age were maintained in ventilated (up to 50 air exchanges/hour) isolator cages (cages with dimensions of 10 ¾″ W×19 ¼″ D×10 ¾″ H, 143 square inch floor space, fitted in Micro-vent 36-cage rat racks; Allentown Inc., Allentown, NJ) containing Aspen Sani chips (pinewood shavings from Harlan) as bedding, and a 14 h light: 10 h dark regimen, at a temperature of 70 F and humidity of 25% to 35%. The mean light intensity in the animal rooms ranged from 22 to 26 ft-candles. Rats were fed ad lib with standard rat diet (8640 Teklad 22/5 Rodent Diet; Harlan) and ad lib tap water for drinking. During the procedures, rats were held in an animal transfer station (AniGard 6VF, The Baker Company, Sanford, ME) that provided an air velocity of about 0.5 inch.

At proestrus as determined by daily vaginal smears, the female rats, (90 days) were pair-mated with male rats (120 days). On the next day, the females were separated and their vaginal smears were examined microscopically and if they were sperm-positive (day 0) the rats were tentatively considered pregnant and then weighed with a digital animal weighing balance to monitor increases in body weight. Vaginal smears were continued for monitoring diestrus status in these rats until day 7. On embryonic day 7 (E-7) these females were weighed to determine if there was a significant increase in (greater than about 10 g) body weight, to confirm pregnancy in sperm-positive females. These pregnant rats were then given daily intraperitoneal injections of any one of the following single chemicals or mixtures with an equal volume of sesame oil (Sigma) on days E-8 through E-14 of gestation [43]. Treatment groups were Control, Pesticide (Permethrin+DEET), Plastics (Bisphenol-A, DBP and DEHP), Dioxin (TCDD), and Jet Fuel (JP8 hydrocarbon). The pregnant female rats treated with various mixtures were designated as the F0 generation. When there was a drop in the litter size and the sex ratio of pups in F1 generation of Plastics group, another treatment group was included with only half the dose of Bisphenol-A, DBP and DEHP and this group was designated ‘Low Dose Plastics’ group. Doses, percent of oral LD50, and sources of chemicals for the compounds are given in Table S1A.

### Breeding for F1, F2, and F3 generations, weaning measures and puberty checks

The offspring of the F0 generation were the F1 generation. Likewise F2 and F3 generation offspring were generated. The breeding used males and females from the same lineage (control or exposure), but did not use any sibling or cousin crosses to avoid inbreeding artifacts. These rats were weaned from their mothers at 21 days of age. At weaning, the following weaning traits were measured; litter size, sex ratio, weaning weight (in grams), and anogenital index (AGI). Anogenital distance (AGD), was measured with a caliper that had an accuracy of 1/100^th^ of a mm. Males have a significantly higher AGD than that of females. Weaning weights of rats were measured by a digital balance. AGI was computed as the AGD in mm (from the ventral edge of the anal opening to the caudal edge of the genital opening) per gram of body weight at weaning. Starting at the age of 30 days for females and 35 days for males, puberty checks were performed. These checks were performed on a daily basis until puberty in each rat was confirmed. Onset of puberty for females was indicated by a clear vaginal opening, and for males it was indicated when the glans penis was able to fully extend free of the preputial fold (balano-preputial separation) [37] (Figure S6).

### Dissection of rats for tissue collection

Both female and male rats of F1, F2 and F3 generation at 90–120 days of age were euthanized by CO_2_ inhalation and cervical dislocation for dissection, collection and examination of tissues including testis, epididymis, and ovary. Body and tissue weights were measured at dissections. Blood samples were collected, allowed to clot, centrifuged and serum samples stored for hormone assays. Tissues were fixed in Bouin's solution (Sigma) and 70% ethanol, then processed for paraffin embedding by standard procedures for histopathology examination. Five-micrometer sections were made and were either unstained or stained with H & E stain.

### TUNEL cell death assay

Testis sections were examined by Terminal deoxynucleotidyl transferase-mediated dUTP nick end labeling (TUNEL) assay (In situ cell death detection kit, Fluorescein, Roche Diagnostics, Mannheim, Germany) as per the manufacturer's protocols. The sections were deparaffinized in xylene, rehydrated through descending series of ethyl alcohols, deionized water and 1× PBS buffer. The sections were deproteinized by incubation at 37°C in 250 ml of 1× PBS buffer containing 150 µl of Fungal Proteinase K (20 mg/ml; Invitrogen, Carlsbad, CA) and washed in 1× PBS buffer. About 20–25 µl of the enzyme-label solution mix was applied to testis sections. Slides were incubated at 37°C for 90 min, washed in fresh 1× PBS buffer for 10 min, mounted with GVA mount and kept at 4°C until examination. Testis sections were examined in a fluorescent microscope in dark to count the number of brightly fluorescing germ cells that are apoptotic.

### Ovarian analysis

Evaluation of adult ovaries: Ovaries taken from rats at the time of sacrifice were fixed, paraffin embedded and sectioned at 5 µm thickness. Every 30th section was collected and hematoxylin/eosin stained. The three stained sections (150 µm apart) through the central portion of the ovary with the largest cross-section were evaluated for number of primordial follicles, developing pre-antral follicles, small antral follicles, large antral follicles, small cystic structures and large cysts. The mean number of each evaluated structure per section was calculated across the three sections. Follicles had to be non-atretic and have the oocyte nucleus visible in the section in order to be counted. Primordial follicles had an oocyte surrounded by a single layer of either squamous or both squamous and cuboidal granulosa cells [44]. Developing pre-antral follicles had one or more complete layers of cuboidal granulosa cells. Small antral follicles had a fluid-filled antrum and a maximum diameter of 51 µm measured across the outermost granulosa cell layer. Large antral follicles had a diameter greater than 51 µm.

### Sperm DNA isolation and methylated DNA immunoprecipitation (MeDIP)

Sperm heads were separated from tails through sonication following previously described protocol (without protease inhibitors) [45] and then purified using a series of washes and centrifugations [46] from a total of nine F3 generation rats per treatment lineage that were 120 days of age. DNA extraction on the purified sperm heads was performed as previously described [5]. Equal concentrations of DNA from individual sperm samples were then used to produce pools of DNA material. Three DNA pools were produced in total per treatment, which contained the same amount of sperm DNA from three animals. Therefore a total of 45 animals were used for building three DNA pools per treatment for the 4 experimental groups plus controls. These DNA pools were then used for methylated DNA immunoprecipitation (MeDIP). MeDIP was performed as follows: 6 µg of genomic DNA was subjected to series of three 20 pulse sonications at 20% amplitude and the appropriate fragment size (200–1000 ng) was verified through 2% agarose gels; the sonicated genomic DNA was resuspended in 350 ul TE and denaturated for 10 min at 95°C and then immediately placed on ice for 5 min; 100 ul of 5× IP buffer (50 mM Na-phosphate pH 7, 700 mM NaCl, 0.25% Triton X-100) was added to the sonicated and denatured DNA. An overnight incubation of the DNA was performed with 5 ug of antibody anti-5-methylCytidine monoclonal from Diagenode S.A (Denville, NJ) at 4°C on a rotating platform. Protein A/G beads from Santa Cruz (Santa Cruz, CA) were prewashed on PBS-BSA 0.1% and resuspended in 40 ul 1× IP buffer. Beads were then added to the DNA-antibody complex and incubated 2 h at 4°C on a rotating platform. Beads bound to DNA-antibody complex were washed 3 times with 1 ml 1× IP buffer; washes included incubation for 5 min at 4°C on a rotating platform and then centrifugation at 6000 rpm for 2 min. Beads-DNA-antibody complex were then resuspended in 250 ul digestion buffer (50 mM Tris HCl pH 8, 10 mM EDTA, 0.5% SDS) and 3.5 ul of proteinase K (20 mg/ml) was added to each sample and then incubated overnight at 55°C on a rotating platform. DNA purification was performed first with phenol and then with chloroform∶isoamyl alcohol. Two washes were then performed with 70% ethanol, 1 M NaCl and glycogen. MeDIP selected DNA was then resuspended in 30 ul TE buffer.

### Tilling array MeDIP-Chip analysis

Roche Nimblegen's Rat DNA Methylation 3×720 K CpG Island Plus RefSeq Promoter Array was used, which contains three identical sub-arrays, with 720,000 probes per sub-array, scanning a total of 15,287 promoters (3,880 bp upstream and 970 bp downstream from transcription start site). Probe sizes range from 50–75 mer in length with the median probe spacing of 100 bp. Three different comparative (MeDIP vs MeDIP) hybridizations experiments were performed for each experimental group versus control, each encompassing DNA samples from 6 animals (3 treatment and 3 control groups) and 3 sub-arrays. MeDIP DNA samples from experimental groups were labeled with Cy3 and MeDIP DNA samples from the control group were labeled with Cy5.

### Bioinformatic and statistic analyses of Chip data

For each comparative hybridization experiment, raw data from both the Cy3 and Cy5 channels were imported into R (R Development Core Team (2010), R: A language for statistical computing, R Foundation for Statistical Computing, Vienna, Austria. ISBN 3-900051-07-0, URL http://www.R-project.org), checked for quality and converted to MA values (M = Cy5-Cy3; A = (Cy5+Cy3)/2). The following normalization procedure was conducted. Within each array, probes were separated into groups by GC content and each group was separately normalized, between Cy3 and Cy5 using the loess normalization procedure. This allowed for GC groups to receive a normalization curve specific to that group. After each array was normalized within array, the arrays were then normalized across arrays using the A quantile normalization procedure.

Following normalization each probe within each array was subjected to a smoothing procedure, whereby the probe's normalized M values were replaced with the median value of all probe normalized M values across all arrays within a 600 bp window. If the number of probes present in the window was less than 3, no value was assigned to that probe. Each probe's A values were likewise smoothed using the same procedure. Following normalization and smoothing each probe's M value represents the median intensity difference between vinclozolin lineage and control lineage of a 600 bp window. Significance was assigned to probe differences between lineage and generation samples by calculating the median value of the intensity differences as compared to a normal distribution scaled to the experimental mean and standard deviation of the normalized M. A Z-score and P-value were computed for each probe from that distribution. The statistical analysis was performed in pairs of comparative IP hybridizations between treatment lineage (T) and control lineage (C) (e.g. T1-C1 and T2-C2; T1-C1 and T3-C3; T2-C2 and T3-C3). In order to assure the reproducibility of the candidates obtained, only the candidates showing significant changes in every one of the paired comparisons were chosen as having a significant change in DNA methylation between each of the experimental group and controls. This is a very stringent approach to select for changes, since it only considers repeated changes in all paired analysis.

Clustered Regions of interest were then determined by combining consecutive probes within 600 bases of each other, and based on whether their mean M values were positive or negative, with significance p-values less than 10^−5^. The statistically significant differential DNA methylated regions were identified and P-value associated with each region presented. Each region of interest was then annotated for gene and CpG content. This list was further reduced to those regions with an average intensity value exceeding 9.5 (log scale) and a CpG density ≥1 CpG/100 bp.

### MeDIP-qPCR confirmation

The MeDIP-Chip differential DNA methylation sites identified were further tested with a quantitative PCR analysis [47], [48]. Real time qPCR quantification of each significant region obtained from the array was performed on MeDIP samples and the values were normalized to the DNA concentration of MeDIP samples measured by picogreen. These qPCR assays were optimized and performed by the Genomics Core Laboratory at the University of Arizona, Tucson, AZ. Three technical replicates of Real Time qPCR reactions were performed for each one of three different MeDIPs per experimental group. Each MeDIP was from pools of sperm DNA samples from three animals. Ct values were obtained and the relative presence of specific DNA amplicons was calculated between control and exposure groups through the equation ‘relative change = 2^−ΔCt^’. Statistical analysis between control and exposure groups was performed with student's t-test and changes with p<0.05 were considered significant. The level of DNA in the pool is a weighted average of all individuals, as previously described, [49].

### Statistical analysis

For statistical analysis, all the data on weaning traits and onset of puberty were averaged for each litter. These averages were used as input in the program GraphPad© Prism 5 statistical analysis program. One-way ANOVA or t-test were used to determine if the data on puberty, number of apoptotic germ cells, number of ovarian follicles from the individual treatment groups differ from those of Control groups with a probability of significance, *p = 0.05*.

## Supporting Information

Figure S1 **Weaning traits including litter size and sex ratio were measured in three generations of rat progeny derived from pregnant F0 females exposed to environmental compounds (Pesticide, Plastics, Dioxin and Jet Fuel).** Litter size and sex ratio were reduced only in Plastics group in F1 generation rats (* p<0.05; **p<0.01).
(PDF)

Figure S2 **Weaning weight measured in three generations of rat offspring derived from pregnant F0 females exposed to environmental compounds (Pesticide, Plastics, Dioxin and Jet Fuel).** Weaning weight increased only in Pesticide group in F2 generation rats.
(PDF)

Figure S3 **Anogenital indexes (AGI) were computed based on anogenital distance and weaning weights in three generations of rat offspring derived from pregnant F0 females exposed to environmental compounds (Pesticide, Plastics, Dioxin and Jet Fuel).** AGI was unaffected in both female and male rats of F1 generations. AGI was reduced in Pesticide and Plastics groups of F2 female rats while it increased in LD Plastics F2 female rats. AGI declined in Pesticide group of F2 male rats while it increased in LD Plastics F2 male rats (* p<0.05; **p<0.01).
(PDF)

Figure S4 **Onset of puberty in female and male rats were investigated in three generations of rat offspring derived from pregnant F0 females exposed to environmental compounds (Pesticide, Plastics, Dioxin and Jet Fuel).** Data from the first two generations are shown. (Puberty data of F3 generation rats are presented in Fig. 1). In the F1 generation, a delayed onset of puberty was recorded in female rats of Plastics group, and male rats of Plastics and Jet Fuel groups. In the F2 generation, an early onset of puberty was found for females rats of Plastics, LD Plastics, Dioxin and Jet Fuel groups and for the male rats of Plastics and Dioxin groups (* p<0.05; **p<0.01; ***p<0.001).
(PDF)

Figure S5 Serum hormone concentrations were measured in the third generation rat offspring derived from pregnant F0 females exposed to environmental compounds (Pesticide, Plastics, Dioxin and Jet Fuel)(* p<0.05; **p<0.01). (A) Serum testosterone concentrations in male rats were severely reduced in Plastics, Dioxin and Jet Fuel groups. (B) Serum progesterone concentrations were unaffected in female rats (C) Serum LH concentrations were unaltered in male rats (D) Serum LH concentrations were not changed in female rats.
(PDF)

Figure S6 Onset of puberty was identified by (A) the opening of vaginal orifice in female rats and (B) the separation of glans penis from the prepuce in male rats.
(PDF)

Table S1 **Doses and Sources of Chemicals used.**
(PDF)

Table S2 **List of rat sperm differential methylation regions (DMR).**
(PDF)

Table S3 **Pathways influenced by genes associated with DMR.**
(PDF)
