# Supplementary figures and images for: Transgenerational Actions of Environmental Compounds on Reproductive Disease and Identification of Epigenetic Biomarkers of Ancestral Exposures
Source: PLoS One. 2012 Feb 28;7(2):e31901. doi: 10.1371/journal.pone.0031901 (PMC3289630; doi:10.1371/journal.pone.0031901)

Supplemental Figure S1. Litter size and sex ratio in F1-F3 generations

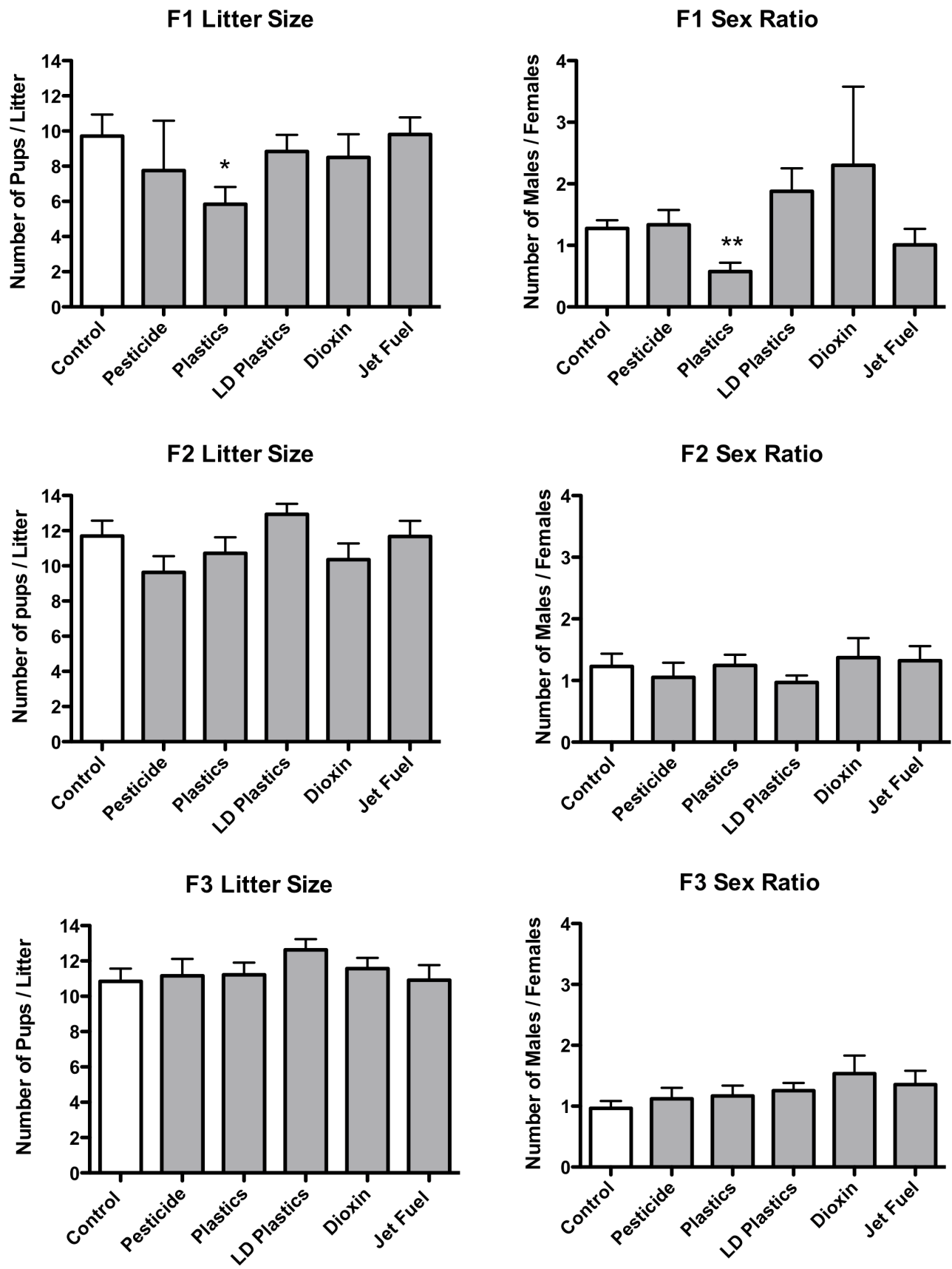

Supplement: Figure S1 — Weaning traits including litter size and sex ratio were measured in three generations of rat progeny derived from pregnant F0 females exposed to environmental compounds (Pesticide, Plastics, Dioxin and Jet Fuel). Litter size and sex ratio were reduced only in Plastics group in F1 generation rats (* p<0.05; **p<0.01). (PDF) [file pone.0031901.s001.pdf]

Supplemental Figure S2. Weaning weights in F1-F3 generations

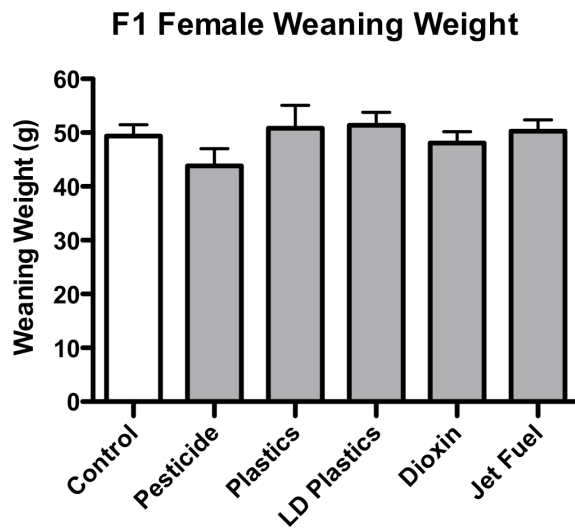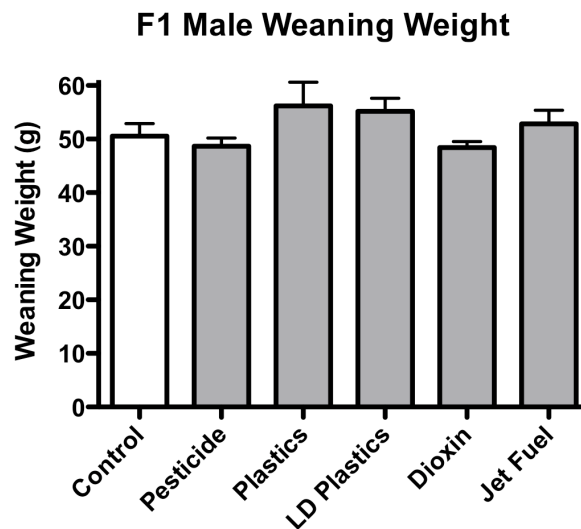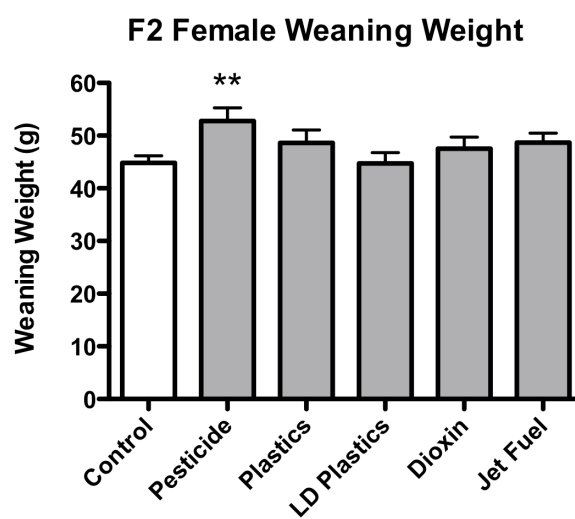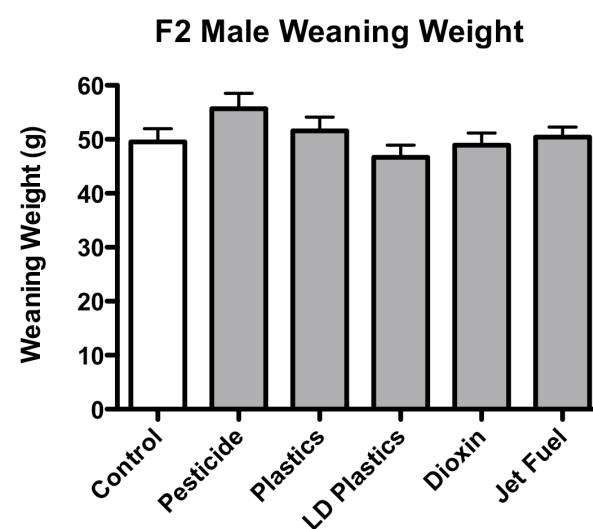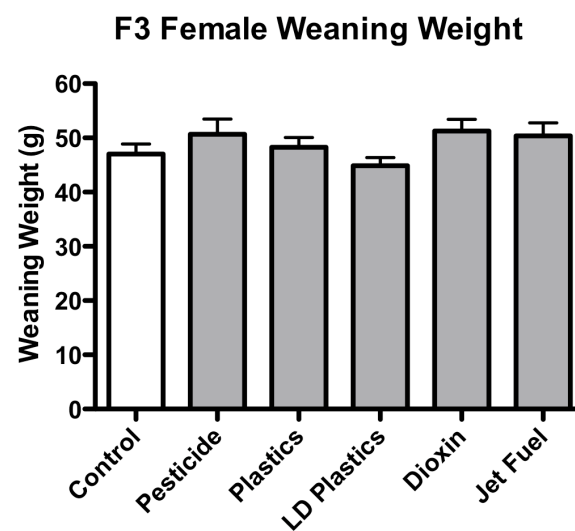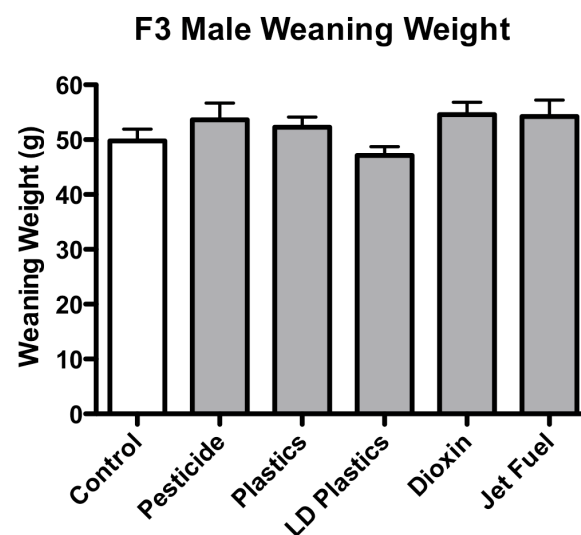

Supplement: Figure S2 — Weaning weight measured in three generations of rat offspring derived from pregnant F0 females exposed to environmental compounds (Pesticide, Plastics, Dioxin and Jet Fuel). Weaning weight increased only in Pesticide group in F2 generation rats. (PDF) [file pone.0031901.s002.pdf]

Supplemental Figure S3. Anogenital Index in F1-F3 generations

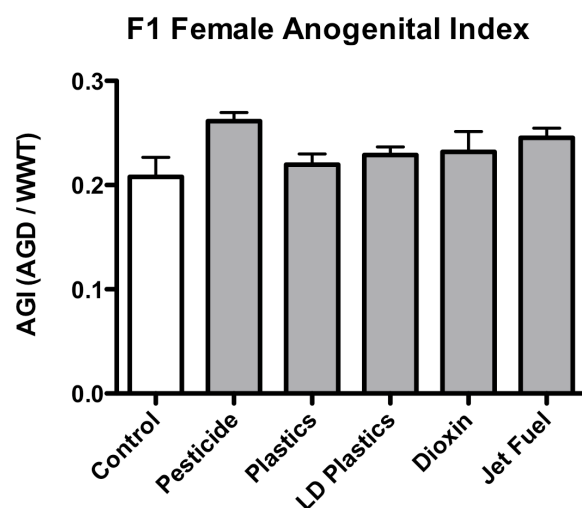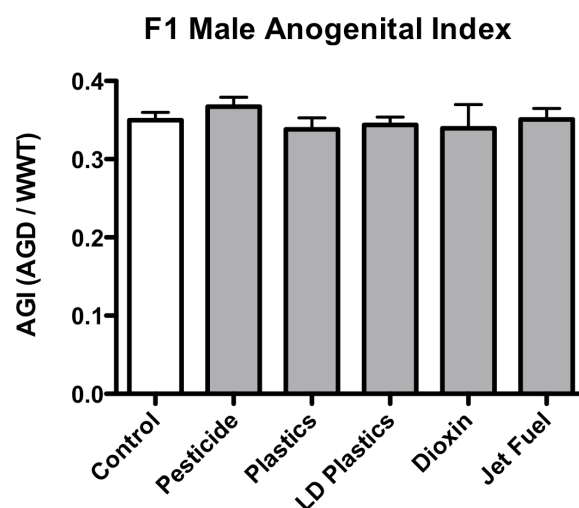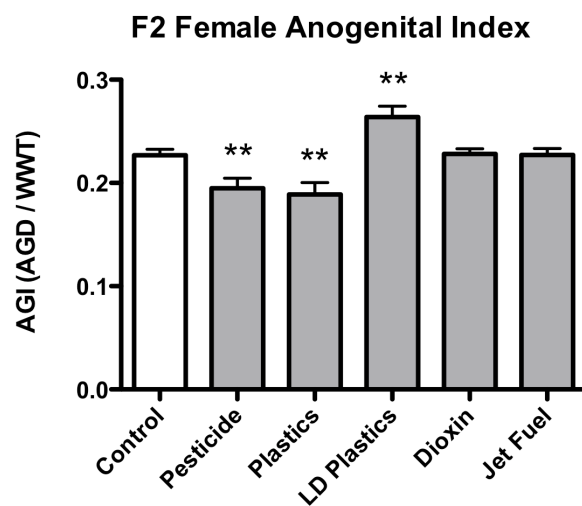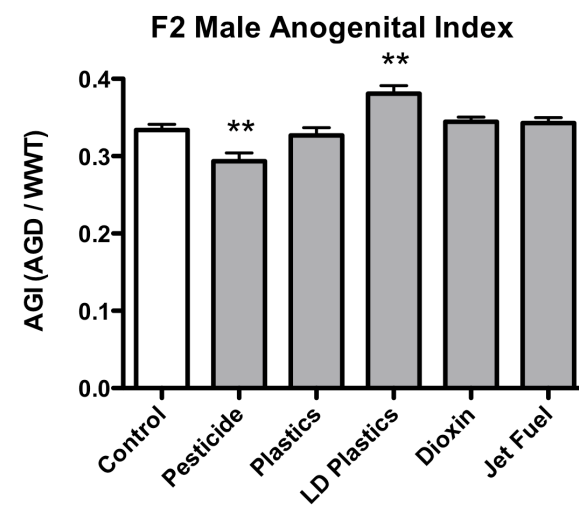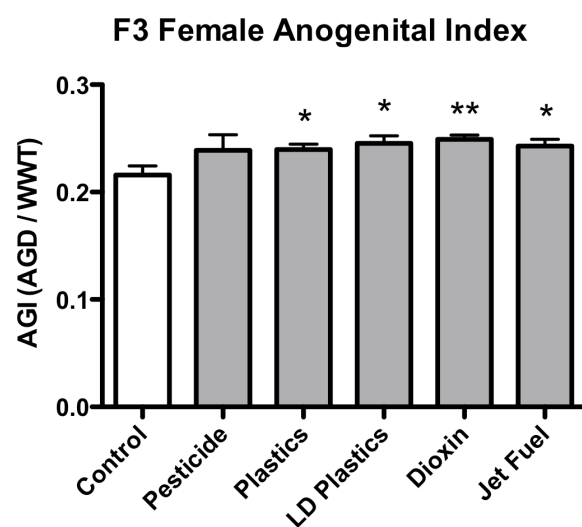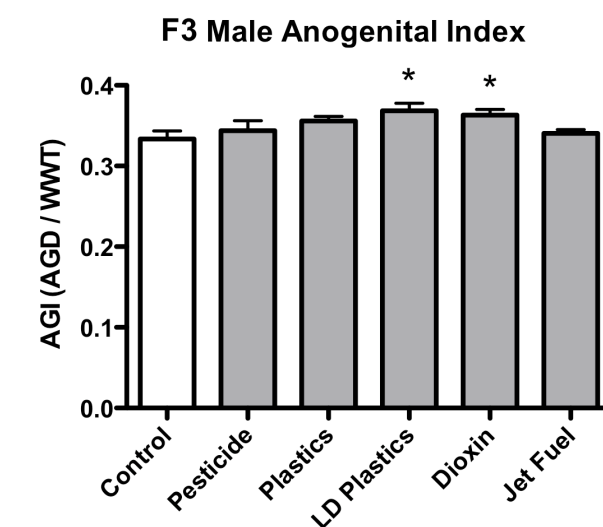

Supplement: Figure S3 — Anogenital indexes (AGI) were computed based on anogenital distance and weaning weights in three generations of rat offspring derived from pregnant F0 females exposed to environmental compounds (Pesticide, Plastics, Dioxin and Jet Fuel). AGI was unaffected in both female and male rats of F1 generations. AGI was reduced in Pesticide and Plastics groups of F2 female rats while it increased in LD Plastics F2 female rats. AGI declined in Pesticide group of F2 male rats while it increased in LD Plastics F2 male rats (* p<0.05; **p<0.01). (PDF) [file pone.0031901.s003.pdf]

Supplemental Figure S4. Puberty Analysis in the F1 and F2 generations

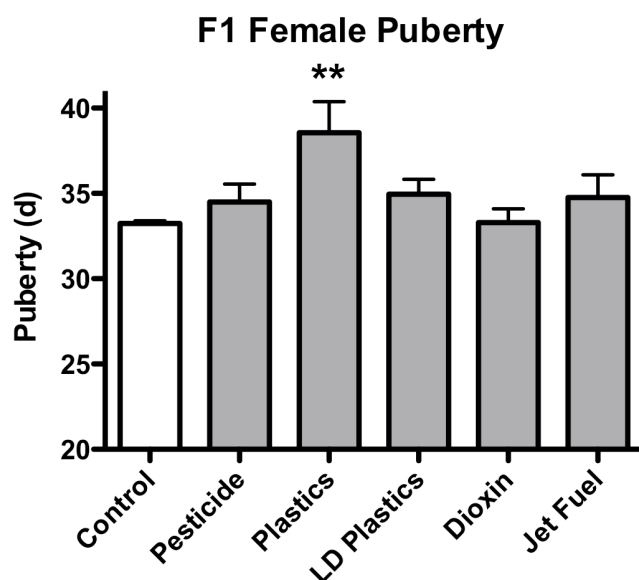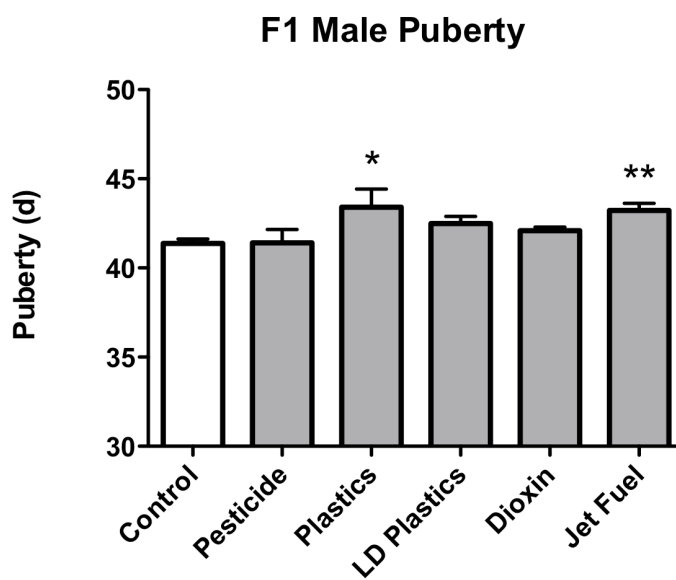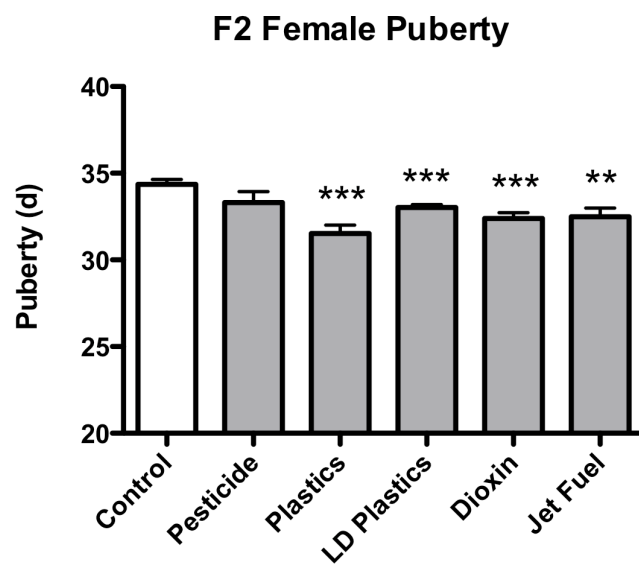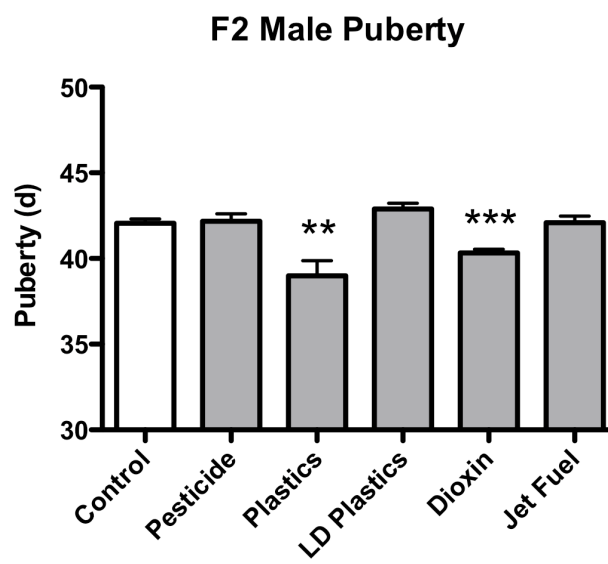

Supplement: Figure S4 — Onset of puberty in female and male rats were investigated in three generations of rat offspring derived from pregnant F0 females exposed to environmental compounds (Pesticide, Plastics, Dioxin and Jet Fuel). Data from the first two generations are shown. (Puberty data of F3 generation rats are presented in Fig. 1). In the F1 generation, a delayed onset of puberty was recorded in female rats of Plastics group, and male rats of Plastics and Jet Fuel groups. In the F2 generation, an early onset of puberty was found for females rats of Plastics, LD Plastics, Dioxin and Jet Fuel groups and for the male rats of Plastics and Dioxin groups (* p<0.05; **p<0.01; ***p<0.001). (PDF) [file pone.0031901.s004.pdf]

Supplemental Figure S6. Analysis of onset of puberty in (A) female and (B) male rats

**(A)**

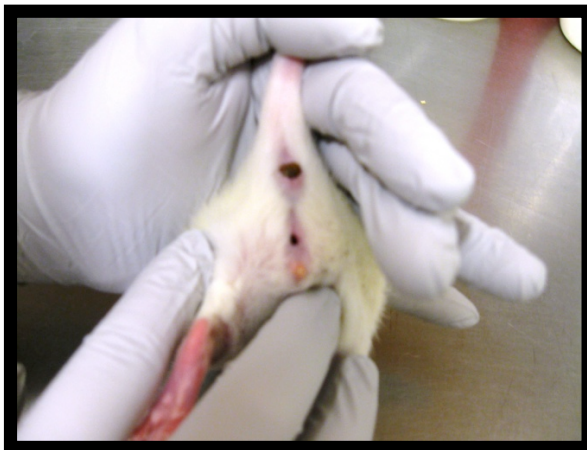

**(B)**

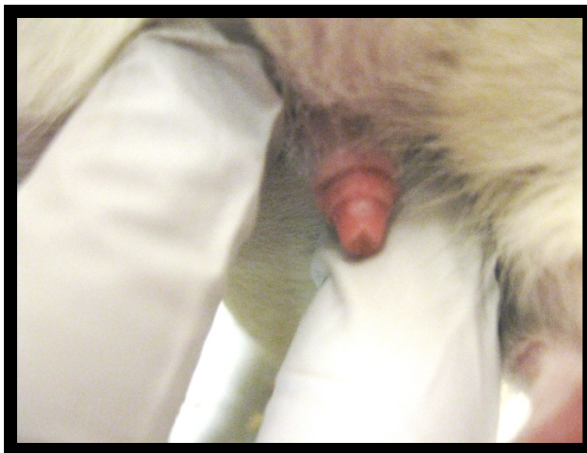

Supplement: Figure S6 — Onset of puberty was identified by (A) the opening of vaginal orifice in female rats and (B) the separation of glans penis from the prepuce in male rats. (PDF) [file pone.0031901.s006.pdf]
