# Supplementary material for: Transgenerational Actions of Environmental Compounds on Reproductive Disease and Identification of Epigenetic Biomarkers of Ancestral Exposures
Source: PLoS One. 2012 Feb 28;7(2):e31901. doi: 10.1371/journal.pone.0031901 (PMC3289630; doi:10.1371/journal.pone.0031901)

Supplemental Figure S5. Serum steroid hormone and LH concentrations in F3 generation rats

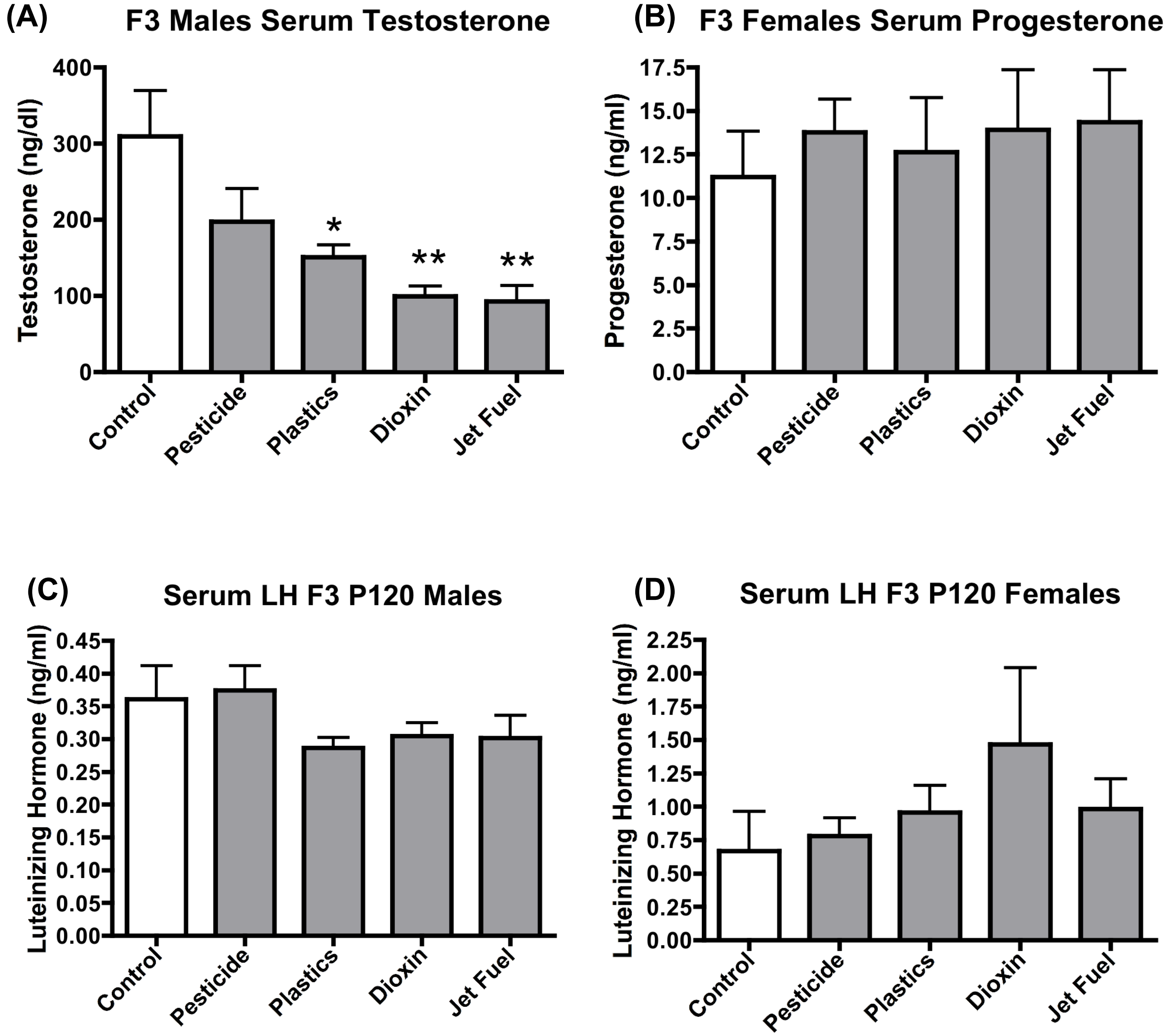

Supplement: Figure S5 — Serum hormone concentrations were measured in the third generation rat offspring derived from pregnant F0 females exposed to environmental compounds (Pesticide, Plastics, Dioxin and Jet Fuel)(* p<0.05; **p<0.01). (A) Serum testosterone concentrations in male rats were severely reduced in Plastics, Dioxin and Jet Fuel groups. (B) Serum progesterone concentrations were unaffected in female rats (C) Serum LH concentrations were unaltered in male rats (D) Serum LH concentrations were not changed in female rats. (PDF) [file pone.0031901.s005.pdf]
