# Supplementary material for: Transgenerational Actions of Environmental Compounds on Reproductive Disease and Identification of Epigenetic Biomarkers of Ancestral Exposures
Source: PLoS One. 2012 Feb 28;7(2):e31901. doi: 10.1371/journal.pone.0031901 (PMC3289630; doi:10.1371/journal.pone.0031901)
Supplement: Table S1 — Doses and Sources of Chemicals used. (PDF) [file pone.0031901.s007.pdf]

Supplemental Table S1A. Doses and Sources of Chemicals used

Chemical	Dose	Source	% Oral LD50 Dose
Control (DMSO, 50%)	100 µl/kg/BW/d	Sigma Aldrich Corp, St. Louis, MO	
Permithrin	150 mg/kg BW/d	Sigma Aldrich Corp, St. Louis, MO	39%
DEET	40 mg/kg BW/d	Chem Service, West Chester, PA	2%
Bisphenol A	50 mg/kg BW/d	Sigma Aldrich Corp, St. Louis, MO	1%
DEHP	750 mg/kg BW/d	Sigma Aldrich Corp, St. Louis, MO	0.025%
DBP	66 mg/kg BW/d	Sigma Aldrich Corp, St. Louis, MO	0.8%
TCDD	100 ng/kg BW/d	Cambridge Isotope Labs, Andover, MA	0.1%
Jet Fuel	500 mg/kg BW/d	Lt Dean Wagner, Dayton, OH	25%

The low dose plastics used 50% the dose listed above for BPA, DEHP and DBP

Supplemental Table S1B. Number of rat litters weaned for three generations.

Group	F1	F2	F3
Control	7	12	25
Pesticides	4	8	13
Plastics	6	14	14
LD Plastics	12	14	19
Dioxin	4	14	14
Jet Fuel	5	18	12
Total	38	80	97

Supplemental Table S1C: Number of male and female rat pups weaned for three generations.

Group	F1 Female/Male	F2 Female/Male	F3 Female/Male
Control	30/38	65/77	131/151
Pesticides	13/18	40/37	70/75
Plastics	21/14	72/78	77/80
LD Plastics	44/61	95/86	111/129
Dioxin	15/20	68/78	84/78
Jet Fuel	26/22	107/103	68/63
Total	149/173	447/459	541/576
