# Supplementary material for: Transgenerational Actions of Environmental Compounds on Reproductive Disease and Identification of Epigenetic Biomarkers of Ancestral Exposures
Source: PLoS One. 2012 Feb 28;7(2):e31901. doi: 10.1371/journal.pone.0031901 (PMC3289630; doi:10.1371/journal.pone.0031901)
Supplement: Table S2 — List of rat sperm differential methylation regions (DMR). (PDF) [file pone.0031901.s008.pdf]

Supplemental Table S2A.
List of rat sperm differential methylation regions (DMR) found in F3-generation after exposure of F0-generation to Pesticide

Gene Symbol	Chr	Start	Stop	Gene ID	min p-value	DMR Cluster Location*	GeneTitle
**Apoptosis**							
Pdcd11	1	252412922	252413522	309458	1.3E-09		programmed cell death 11
Siva1	6	137630549	137631439	362791	1.2E-12		SIVA1, apoptosis-inducing factor
Card10	7	116767137	116768035	315120	8.7E-08	chr7:116.3	caspase recruitment domain family, member 10
Tnfrsf12a	10	12940938	12942568	302965	5.2E-23		tumor necrosis factor receptor superfamily, member 12a
Higd2a	17	16084041	16084831	290999	2.4E-14		HIG1 hypoxia inducible domain family, member 2A
Tnfaip8	18	45084538	45085228	307428	1.8E-13		tumor necrosis factor, alpha-induced protein 8
**Cell Cycle**							
Cep55	1	242381886	242382486	294074	3.8E-15		centrosomal protein 55kDa
Cdk9	3	11677067	11677767	362110	4.1E-08		cyclin-dependent kinase 9
Cdk5	4	6116554	6117864	140908	9.6E-10		cyclin-dependent kinase 5
Cdk2	7	2006927	2007527	362817	6.1E-09	chr7:1.25	cyclin dependent kinase 2
Dock6	8	20894272	20895262	367039	4.2E-20	chr8:20	dedicator of cytokinesis 6
Gas8	19	53834037	53834839	361438	2.4E-09		growth arrest specific 8
Pak3	X	34841200	34842210	29433	4.9E-10		p21 protein (Cdc42/Rac)-activated kinase 3
**Cytoskeleton-ECM**							
Clec11a	1	94792412	94793122	29313	8.0E-08	chr1:92.8	C-type lectin domain family 11, member a
Actn3	1	207492038	207492749	171009	7.4E-10		actinin alpha 3
Add3	1	259452730	259453611	25230	2.7E-13		adducin 3 (gamma)
Lmo1	1	166741881	166742882	245979	3.4E-08		LIM domain only 1
Krtcap2	2	181416132	181417032	295243	1.2E-10	chr2:180.7	keratinocyte associated protein 2
Actl6a	2	118918849	118919939	361925	9.2E-14		actin-like 6A
Flg	2	186309317	186310200	24641	5.6E-12		filaggrin
Cercam	3	8877244	8878133	296616	2.7E-10		cerebral endothelial cell adhesion molecule
LOC313641	5	156333892	156334783	313641	1.6E-10		perlecan
Tpm2	5	60003516	60004592	500450	2.1E-09		tropomyosin 2, beta
Icam5	8	20966220	20967039	313785	7.6E-09	chr8:20	intercellular adhesion molecule 5, telencephalin
Cspg5	8	114620901	114621591	50568	7.1E-09	chr8:114.3	chondroitin sulfate proteoglycan 5
LOC100363366	8	30972488	30973193	100363366	1.0E-12		amyloid beta (A4) precursor-like protein 2-like
Tpm1	8	71356826	71357726	24851	2.3E-13		tropomyosin 1, alpha
Lasp1	10	86551715	86552513	29278	5.6E-08		LIM and SH3 protein 1
Mapt	10	93411242	93412032	29477	1.9E-07		microtubule-associated protein tau
Mprip	10	45975563	45976456	116504	7.5E-16		myosin phosphatase Rho interacting protein
Snip	10	86320851	86321529	56029	1.0E-10		SNAP25-interacting protein
Snip	10	86323498	86324403	56029	9.7E-10		SNAP25-interacting protein
Ncam2	11	20423008	20423897	288280	7.4E-08		neural cell adhesion molecule 2
Vcl	15	3575689	3576884	305679	1.1E-11		vinculin
Fat1	16	50588806	50589905	83720	2.4E-13		FAT tumor suppressor homolog 1 (Drosophila)
Tubb2a	17	37138997	37140410	498736	2.5E-17		tubulin, beta 2a
Vim	17	87847877	87848477	81818	1.8E-09		vimentin
Pcdhb15	18	30286538	30287330	291646	1.1E-11		protocadherin beta 15
Cdh11	19	2225447	2226147	84407	3.1E-08		cadherin 11
Lrg1	Un/9	25672410	25673300	367455	2.1E-15		leucine-rich alpha-2-glycoprotein 1
Plin5	Un/	25672410	25673300	501283	2.1E-15		perilipin 5

	9							
**Development**								
Dmpk	1	78450272	78451687	308405	2.0E-23	chr1:78.1	dystrophia myotonica-protein kinase
Ggn	1	84303670	84304575	292765	1.7E-07	chr1:83.5	gametogenetin
Ttyh1	1	69482346	69483230	292597	5.4E-13		tweety homolog 1 (Drosophila)
Six5	1	78450272	78451687	308406	2.0E-23		SIX homeobox 5
Usmg5	1	252412922	252413522	171069	1.3E-09		up-regulated during skeletal muscle growth 5 homolog (mouse)
Dkk2	2	229541018	229541911	295445	1.4E-09		dickkopf homolog 2 (Xenopus laevis)
Sv2c	2	26554389	26554989	29643	2.6E-07		synaptic vesicle glycoprotein 2c
Disp2	3	105283894	105284775	311324	7.9E-09		dispatched homolog 2 (Drosophila)
Lhx6	3	15214211	15214897	311901	5.8E-08		LIM homeobox 6
Ntng2	3	8228607	8229412	311836	3.4E-18		netrin G2
Qser1	3	90155661	90156556	311266	1.4E-09		glutamine and serine rich 1
SPATA2	3	158640784	158641479	114210	1.1E-14		spermatogenesis associated 2
SMO	4	56653034	56653704	25273	1.8E-13	chr4:56.25	smoothened homolog (Drosophila)
Lbx2	4	117298557	117299157	500224	9.8E-11	chr4:116.7	ladybird homeobox 2
Bspry	5	79471177	79472257	64027	1.5E-08	chr5:79.4	B-box and SPRY domain containing
Vwa1	5	172630371	172631649	298683	1.5E-09	chr5:172	von Willebrand factor A domain containing 1
Npc2	6	108814526	108815606	286898	1.1E-45	chr6:107.8	Niemann-Pick disease, type C2
Bre	6	24791954	24793149	362704	2.0E-09		brain and reproductive organ-expressed protein
Pmel	7	2006927	2007527	362818	6.1E-09		premelanosome protein
Trps1	7	87076226	87077245	299897	3.5E-15		trichorhinophalangeal syndrome I homolog (human)
Tmie	8	115269400	115270189	501061	5.3E-13	chr8:114.3	transmembrane inner ear
Mll1	8	47836284	47837266	315606	1.4E-08		myeloid/lymphoid or mixed-lineage leukemia 1
Aamp	9	73610303	73611481	301512	3.2E-08		angio-associated, migratory cell protein
Nlgn2	10	56675163	56676245	117096	9.4E-13	chr10:55.2	neuroligin 2
Nlgn2	10	56678345	56679370	117096	4.8E-11	chr10:55.2	neuroligin 2
Hoxb6	10	85032294	85033194	497986	5.8E-18	chr10:84	homeo box B6
Auts2	12	25400693	25401534	498173	2.4E-14		autism susceptibility candidate 2
Pbx1	13	84115851	84116728	304947	3.6E-09		pre-B-cell leukemia homeobox 1
Myog	13	47243170	47244059	29148	3.9E-08		myogenin
Pinx1	15	43125066	43125666	305963	1.1E-14		PIN2-interacting protein 1
Unc13a	16	18853366	18853966	64829	2.4E-08	chr16:17.8	unc-13 homolog A (C. elegans)
Irx2	17	746309	746989	306657	8.4E-20		iroquois homeobox 2
Irx5	19	15751735	15752521	498918	1.2E-09		iroquois homeobox 5
Nodal	20	29546601	29547322	294503	1.9E-18		nodal homolog (mouse)
Bex2	X	123323586	123324186	363498	7.7E-09		brain expressed X-linked 2
Bex4	X	123441916	123442626	501624	8.9E-09		brain expressed gene 4
**DNA Repair**								
Rad54l2	8	111984966	111985566	363135	3.7E-07		Rad54 like 2 (S. cerevisiae)
**Electron Transport**								
Cox6b2	1	67810664	67811751	654441	3.1E-08	chr1:66.9	cytochrome c oxidase subunit VIb polypeptide 2
Prdx5	1	209585932	209587138	113898	7.6E-17		peroxiredoxin 5
Cyp24a1	3	161553659	161554660	25279	1.3E-13		cytochrome P450, family 24, subfamily a, polypeptide 1
Cyp4f5	7	13119902	13120699	286905	6.6E-25		cytochrome P450, family 4, subfamily f, polypeptide 5
Nxnl1	16	18769847	18771129	306342	5.1E-13	chr16:17.8	nucleoredoxin-like 1
**Epigenetics**								
Suv420h2	1	67823420	67824426	308345	5.8E-10	chr1:66.9	suppressor of variegation 4-20 homolog 2 (Drosophila)
Eed	1	146589901	146590900	293104	7.2E-08		embryonic ectoderm development
Smarca2	1	230016032	230016827	361745	2.8E-11		SWI/SNF related, matrix associated, regulator of chromatin
CXXC4	2	231704355	231705138	83824	3.5E-08		CXXC finger 4
Setd5	4	148949953	148951038	297514	3.4E-12		SET domain containing 5
H1f0	7	117001353	117002342	24437	1.3E-12	chr7:116.3	H1 histone family, member 0

Cbx6	7	118069182	118069782	315136	3.4E-10		chromobox homolog 6
Satb2	9	55824749	55825838	501145	3.2E-13		SATB homeobox 2
Kdm6b	10	56204571	56206172	363630	1.4E-16	chr10:55.2	lysine (K)-specific demethylase 6B
Phf15	10	37379987	37381795	303113	1.2E-13		PHD finger protein 15
Tbx2	10	74084425	74085225	303398	5.3E-33		T-box 2
ASMT	12	16815952	16816847	246281	8.8E-18	chr12:15.8	acetylserotonin O-methyltransferase
Asmtl	12	16824308	16825214	288527	1.0E-09		acetylserotonin O-methyltransferase-like
Kdm2b	12	34672184	34673309	304495	3.8E-14		lysine (K)-specific demethylase 2B
Gadd45g	17	19231639	19232721	291005	1.3E-18		growth arrest and DNA-damage-inducible, gamma
Hist1h2bn	17	50352153	50352753	291157	3.1E-17		histone cluster 1, H2bn
Hdac3	18	30875498	30876184	84578	3.2E-14		histone deacetylase 3
**Golgi Apparatus**							
B3gnt8	1	80834920	80835615	308440	4.7E-09	chr1:78.1	UDP-GlcNAc:betaGal beta-1,3-N-acetylglucosaminyltransferase
Mgat4b	10	35775455	35776055	303100	7.2E-12		mannosyl (alpha-1,3-)-glycoprotein beta-1,4-N-acetylglucosamin
**Growth Factors**							
Gdnf	2	57403318	57404513	25453	3.4E-22		glial cell derived neurotrophic factor
MDK	3	76310613	76312125	81517	1.4E-13		midkine
Sspo	4	76528498	76529282	474348	3.1E-12		SCO-spondin
Ntf3	4	162504664	162505639	81737	3.7E-12		neurotrophin 3
WNT10B	7	137545980	137547070	315294	1.3E-13	chr7:136.9	wingless-type MMTV integration site family, member 10B
Dlk2	9	10243248	10244843	316232	4.1E-07	chr9:9.55	delta-like 2 homolog (Drosophila)
VEGFA	9	10521846	10522726	83785	1.8E-07	chr9:9.55	vascular endothelial growth factor A
Wnt3	10	92946863	92947763	24882	9.3E-11		wingless-type MMTV integration site family, member 3
Sept5	11	84364223	84365013	116728	2.4E-08		septin 4
Vgf	12	20905109	20906021	29461	4.9E-10		VGF nerve growth factor inducible
Hgfac	14	81353598	81354616	58947	4.2E-15		hepatocyte growth factor activator
Sept11	14	16548270	16549455	305227	9.4E-19		septin 11
LTB	20	3667367	3668263	361795	4.9E-07	chr20:3.6	lymphotoxin beta (TNF superfamily, member 3)
**Immune Response**							
Igsf10	2	148733771	148734471	310448	1.1E-10		immunoglobulin superfamily, member 10
Cd82	3	77875422	77876242	83628	1.0E-10		Cd82 molecule
Cd276	8	62361422	62362307	315716	1.5E-11		Cd276 molecule
Spag7	10	57548762	57549583	303260	4.8E-07	chr10:55.2	sperm associated antigen 7
Spag7	10	57549874	57551068	303260	1.2E-13	chr10:55.2	sperm associated antigen 7
Fcgr2a	13	86913190	86913875	116591	1.7E-13		Fc fragment of IgG, low affinity IIa, receptor (CD32)
RT1-CE7	20	3416588	3417188	368153	8.1E-18	chr20:2.4	RT1 class I, locus CE7
RT1_CE13	20	89064	90548	414790	1.1E-12		RT1 class I, locus CE13
**Metabolism & Transport**							
Apoe	1	79005645	79006565	25728	2.3E-12	chr1:78.1	apolipoprotein E
Syt3	1	94866084	94867099	25731	2.5E-10	chr1:92.8	synaptotagmin III
Sult2b1	1	96264926	96265526	292915	2.2E-06	chr1:92.8	sulfotransferase family, cytosolic, 2B, member 1
Scd	1	249358774	249359374	83792	6.1E-07		stearoyl-CoA desaturase (delta-9-desaturase)
Syt7	1	212877078	212877873	59267	9.0E-09		synaptotagmin VII
Dhdpsl	1	248805982	248806667	293949	2.2E-06		dihydrodipicolinate synthase-like, mitochondrial
Fah	1	140875689	140876469	29383	2.7E-13		fumarylacetoacetate hydrolase
Slc1a1	1	232487265	232487865	25550	2.8E-11		solute carrier family 1 (neuronal/epithelial high affinity glutamate
Kcnn3	2	181715756	181716441	54263	4.8E-13	chr2:180.7	potassium intermediate/small conductance calcium-activated
Slc25a31	2	127697443	127698323	689108	1.2E-10		solute carrier family 25 (mitochondrial

							carrier; adenine nucleotide
Slc30a5	2	31636928	31637528	294698	2.4E-09		solute carrier family 30 (zinc transporter), member 5
Slc7a14	2	116461907	116462597	499587	1.4E-09		solute carrier family 7 (cationic amino acid transporter
Acoxl	3	115365654	115366254	296138	2.2E-09		acyl-Coenzyme A oxidase-like
Clstn3	4	160665157	160666372	171393	1.1E-10	chr4:160.3	calsyntenin 3
Gapdh	4	161285353	161286243	24383	3.7E-06	chr4:160.3	glyceraldehyde-3-phosphate dehydrogenase
Mat2a	4	105744927	105745527	171347	6.9E-15		methionine adenosyltransferase II, alpha
Magi2	4	11440081	11441061	113970	3.7E-15		membrane associated guanylate kinase, WW and PDZ domain
Sh3bgrl3	5	152877492	152878472	298544	1.3E-10	chr5:151.9	SH3 domain binding glutamic acid-rich protein-like 3
Echdc2	5	129265151	129266066	298381	7.1E-09		enoyl Coenzyme A hydratase domain containing 2
Clstn1	5	166671398	166672378	313717	1.5E-16		calsyntenin 1
Decr1	5	30519336	30520146	117543	2.3E-07		2,4-dienoyl CoA reductase 1, mitochondrial
Pusl1	5	172749107	172749787	362681	1.4E-10		pseudouridylate synthase-like 1
Rbks	6	24791954	24793149	362706	2.0E-09		ribokinase
Srd5a2	6	21452565	21453390	64677	3.8E-09		steroid-5-alpha-reductase, alpha polypeptide 2 (3-oxo-5 alpha-
Pygl	6	92340725	92341524	64035	2.3E-08		phosphorylase, glycogen, liver
Rtn1	6	94681088	94681888	116644	1.3E-12		reticulon 1
SLC39A3	7	10171471	10172556	314637	5.2E-08	chr7:9.6	solute carrier family 39 (zinc transporter), member 3
Cacnb3	7	137383447	137384546	25297	2.1E-10	chr7:136.9	calcium channel, voltage-dependent, beta 3 subunit
Carm1	8	20650587	20651612	363026	8.2E-15	chr8:20	coactivator-associated arginine methyltransferase 1
Pkm2	8	63486309	63487492	25630	2.5E-11		pyruvate kinase, muscle
Stt3a	8	38016123	38016924	500972	1.4E-10		STT3, subunit of the oligosaccharyltransferase complex
Accn4	9	74724160	74724955	63882	2.2E-10		amiloride-sensitive cation channel 4, pituitary
Gnptg	10	14488661	14489341	287134	9.5E-11	chr10:14.1	N-acetylglucosamine-1-phosphate transferase, gamma subunit
Plscr3	10	56687180	56687875	360549	1.4E-09	chr10:55.2	phospholipid scramblase 3
KCNH4	10	89696676	89697576	114032	2.2E-08		potassium voltage-gated channel, subfamily H (eag-related)
Slc9a3r1	10	105236801	105237401	59114	8.0E-08		solute carrier family 9 (sodium/hydrogen exchanger), member 3
Clcn2	11	82426660	82427380	29232	3.9E-07	chr11:81.5	chloride channel 2
Clcn2	11	82429579	82430269	29232	1.0E-08	chr11:81.5	chloride channel 2
Atp6v1a	11	58125901	58126716	685232	2.1E-09		ATPase, H+ transporting, lysosomal V1 subunit A
Acads	12	42765275	42765875	64304	1.1E-11	chr12:42.2	acyl-Coenzyme A dehydrogenase, C-2 to C-3 short chain
Gltp	12	43194112	43195012	288707	7.0E-08	chr12:42.2	glycolipid transfer protein
Mepce	12	19853018	19853828	304361	7.0E-11		methylphosphate capping enzyme
Nmnat2	13	67969800	67970695	289095	2.4E-23		nicotinamide nucleotide adenylyltransferase 2
Tomm40b	13	87111787	87112902	304971	5.8E-16		translocase of outer mitochondrial membrane 40 homolog B
Rhbdd3	14	85758629	85759334	289753	1.7E-09		rhomboid domain containing 3
Enoph1	14	10859038	10860028	305177	1.6E-18		enolase-phosphatase 1
Qdpr	14	70741367	70742367	64192	3.7E-11		quinoid dihydropteridine reductase
Dpysl2	15	46412626	46413517	25416	1.6E-11		dihydropyrimidinase-like 2
Slc6a7	18	56976220	56977132	117100	1.8E-09		solute carrier family 6 (neurotransmitter transporter, L-proline)
Best2	19	24802086	24803166	364973	2.2E-14	chr19:24.1	bestrophin 2
Gpt2	19	22914732	22915711	307759	1.2E-08		glutamic pyruvate transaminase

							(alanine aminotransferase) 2
Gstt2	20	13218263	13218863	29487	2.7E-07		glutathione S-transferase, theta 2
Tap1	20	4789651	4790543	24811	2.9E-10		transporter 1, ATP-binding cassette, sub-family B (MDR/TAP)
**Proteolysis**							
Ctsd	1	202632820	202633700	171293	8.6E-21		cathepsin D
Psma1	1	172248407	172249402	29668	4.0E-16		proteasome (prosome, macropain) subunit, alpha type 1
Serpinh1	1	156673475	156674287	29345	1.9E-07		serine (or cysteine) peptidase inhibitor, clade H, member 1
Ubxn11	5	152877492	152878472	192207	1.3E-10		UBX domain protein 11
Ubxn2b	5	19629204	19629903	312965	1.3E-12		UBX domain protein 2B
Adamts15	7	10873782	10874784	300474	8.7E-17	chr7:9.6	ADAM metallopeptidase with thrombospondin type 1 motif, 15
Prssl1	7	11424819	11425640	408241	5.9E-15		protease, serine-like 1
Xpnpep3	7	119728411	119729114	685823	4.7E-07		X-prolyl aminopeptidase (aminopeptidase P) 3, putative
Ubl5	8	19661648	19662660	500954	3.3E-08		ubiquitin-like 5
AMZ2	10	98899341	98900231	360650	1.5E-07		archaelysin family metallopeptidase 2
Tmub2	10	91364446	91365238	303567	2.4E-07		transmembrane and ubiquitin-like domain containing 2
Mmp17	12	28171455	28172150	288626	2.6E-13	chr12:27.2	matrix metallopeptidase 17
Mmp2	19	15274320	15274920	81686	3.5E-10		matrix metallopeptidase 2
Psmb8	20	4789651	4790543	177046	2.9E-10	chr20:3.6	proteasome (prosome, macropain) subunit, beta type 8 (large
Pi16	20	7642807	7643887	294312	5.6E-12		peptidase inhibitor 16
**Receptors & Binding Proteins**							
Pvrl2	1	79058741	79060044	308417	1.2E-14	chr1:78.1	poliovirus receptor-related 2
Nccrp1	1	83656823	83657423	292755	1.5E-07	chr1:83.5	non-specific cytotoxic cell receptor protein 1 homolog (zebrafish)
Olr168	1	162485084	162485684	293315	5.0E-07		olfactory receptor 168
P2RY6	1	158376135	158377025	117264	1.0E-13		pyrimidinergic receptor P2Y, G-protein coupled, 6
Osbp	1	214811944	214812740	365410	4.2E-13		oxysterol binding protein
Ranbp3l	2	58725068	58726193	294789	1.0E-11		RAN binding protein 3-like
Olr414	3	163798239	163798839	56821	6.5E-07		olfactory receptor 414
Spsb2	4	160931316	160932317	297592	6.6E-23	chr4:160.3	splA/ryanodine receptor domain and SOCS box containing 2
Sec23a	6	79706895	79707495	58817	1.6E-08		Sec23 homolog A (S. cerevisiae)
KISS1R	7	11291511	11292218	78976	5.5E-10	chr7:9.6	KISS1 receptor
Igfbp2	9	71966094	71967073	25662	2.2E-09		insulin-like growth factor binding protein 2
Olr1415	10	44132028	44132628	405059	1.1E-06		olfactory receptor 1415
Il1rap	11	76225521	76226241	25466	5.2E-08		interleukin 1 receptor accessory protein
Pvrl3	11	55842225	55843130	288124	2.4E-08		poliovirus receptor-related 3
IL3RA	12	16829845	16831365	246144	2.6E-09	chr12:15.8	interleukin 3 receptor, alpha
Opn3	13	91426959	91427559	498289	8.1E-12		opsin 3
Aebp1	14	86524798	86525695	305494	1.2E-07		AE binding protein 1
Heatr1	17	68654760	68655860	361262	3.9E-09		HEAT repeat containing 1
Necab2	19	49691211	49692111	170928	2.3E-11		N-terminal EF-hand calcium binding protein 2
Prrt1	20	4220107	4221198	406167	7.8E-20	chr20:3.6	proline-rich transmembrane protein 1
Olr1687	20	4259416	4260219	309574	3.1E-07	chr20:3.6	olfactory receptor 1687
Olr1684	20	3497439	3498621	294151	1.8E-10		olfactory receptor 1684
**Signaling**							
Brsk1	1	67876013	67876613	499073	3.0E-08	chr1:66.9	BR serine/threonine kinase 1
Rabac1	1	80274216	80275308	83583	1.3E-14	chr1:78.1	Rab acceptor 1 (prenylated)
Inppl1	1	159291743	159292748	65038	7.0E-12		inositol polyphosphate phosphatase-like 1
Calca	1	172690039	172691119	24241	9.8E-19		calcitonin-related polypeptide alpha
Shank1	1	94792412	94793122	78957	8.0E-08		SH3 and multiple ankyrin repeat domains 1
Cnksr3	1	37957713	37958623	308113	9.8E-18		Cnksr family member 3

Gng8	1	77218978	77219578	245986	3.0E-21		guanine nucleotide binding protein (G protein), gamma 8
Mrgprg	1	204155989	204156589	309133	2.2E-10		MAS-related GPR, member G
Cks1b	2	181615695	181616678	499655	3.2E-11	chr2:180.7	CDC28 protein kinase regulatory subunit 1B
RAB13	2	182460590	182461570	81756	7.6E-27	chr2:180.7	RAB13, member RAS oncogene family
Shc1	2	181615695	181616678	85385	3.2E-11		SHC (Src homology 2 domain containing) transforming protein 1
Ankrd34a	2	191395150	191396232	295283	5.7E-20		ankyrin repeat domain 34A
ANP32E	2	190715161	190716362	361999	5.7E-18		acidic (leucine-rich nuclear phosphoprotein 32 family, member E
Egflam	2	56666235	56667515	365691	3.6E-11		EGF-like, fibronectin type III and laminin G domains
Notch2	2	192855441	192856541	29492	1.5E-19		Notch homolog 2 (Drosophila)
Ywhab	3	154925245	154926019	56011	2.1E-11	chr3:154.2	tyrosine 3-monooxygenase/tryptophan 5-monooxygenase
Tyro3	3	106308759	106310151	25232	1.3E-08		TYRO3 protein tyrosine kinase
Ptprz1	4	49267340	49268020	25613	1.7E-09		protein tyrosine phosphatase, receptor-type, Z polypeptide 1
Acap3	5	172749107	172749787	313772	1.4E-10	chr5:172	ArfGAP with coiled-coil, ankyrin repeat and PH domains 3
LOC683719	5	86273089	86273964	683719	1.2E-11		similar to RAS and EF hand domain containing
Lpar1	5	76571751	76572551	116744	2.0E-13		lysophosphatidic acid receptor 1
Pink1	5	157102069	157102960	298575	4.5E-09		PTEN induced putative kinase 1
Akap6	6	73042917	73043604	64553	9.4E-61		A kinase (PRKA) anchor protein 6
Itga7	7	2229865	2231454	81008	4.2E-11	chr7:1.25	integrin, alpha 7
Palm	7	11424819	11425640	170673	5.9E-15	chr7:9.6	paralemmin
Shc2	7	11583824	11584614	314612	2.4E-16	chr7:9.6	SHC (Src homology 2 domain containing) transforming protein 2
Grina	7	114277254	114278461	266668	2.9E-19	chr7:114	glutamate receptor, ionotropic, N-methyl D-aspartate-associated
Mapk11	7	127449653	127450253	689314	2.4E-17	chr7:126.8	mitogen-activated protein kinase 11
MAPK8IP2	7	127772817	127773697	315220	3.0E-08	chr7:126.8	mitogen-activated protein kinase 8 interacting protein 2
Shank3	7	127816023	127816717	59312	1.7E-26	chr7:126.8	SH3 and multiple ankyrin repeat domains 3
Itga5	7	142276341	142277241	315346	5.0E-11	chr7:141.3	integrin, alpha 5 (fibronectin receptor, alpha polypeptide)
Anp32a	8	66488052	66489373	25379	1.6E-08		acidic (leucine-rich) nuclear phosphoprotein 32 family, member A
Calml4	8	67005516	67006116	691455	1.7E-25		calmodulin-like 4
Snx33	8	60673757	60674357	315696	1.4E-27		sorting nexin 33
Efnb3	10	56378095	56379587	360546	2.0E-28	chr10:55.2	ephrin B3
Anxa6	10	40438545	40439245	79125	7.7E-11		annexin A6
PLEKHH3	10	90191441	90192426	360634	2.2E-10		pleckstrin homology domain containing, family H (with MyTH4
Ywhae	10	63072423	63073223	29753	9.8E-07		tyrosine 3-monooxygenase/tryptophan 5-monooxygenase
Camk2n2	11	82519950	82520632	59314	4.5E-09	chr11:81.5	calcium/calmodulin-dependent protein kinase II inhibitor 2
Akap17a	12	16824308	16825214	288526	1.0E-09	chr12:15.8	A kinase (PRKA) anchor protein 17A
Gnb2	12	19601090	19602388	81667	8.9E-22		guanine nucleotide binding protein(G protein),beta polypeptide 2
Ptprg	15	15129826	15130718	171357	1.2E-07		protein tyrosine phosphatase, receptor type, G
Dda1	16	18640032	18640843	688813	1.8E-08	chr16:17.8	DET1 and DDB1 associated 1
Gtpbp3	16	18656594	18657490	290633	2.7E-08	chr16:17.8	GTP binding protein 3
Dusp26	16	64958383	64959068	306527	1.3E-09		dual specificity phosphatase 26 (putative)
Psd2	18	28594728	28596406	307500	2.5E-07		pleckstrin and Sec7 domain containing 2
Pdp2	19	31389541	31390235	246311	2.4E-09		pyruvate dehyrogenase phosphatase catalytic subunit 2

Ppp1r10	20	2984086	2984883	65045	2.8E-26		protein phosphatase 1, regulatory subunit 10
Gucy1a2	Un	47455432	47456222	66012	8.1E-11		guanylate cyclase 1, soluble, alpha 2
Ptprs	Un	25300105	25300805	25529	2.5E-16		protein tyrosine phosphatase, receptor type, S
Gprasp1	X	1331319	1331919	171407	4.1E-08		G protein-coupled receptor associated sorting protein 1
**Transcription**							
Mypop	1	78344996	78345976	499090	8.1E-08	chr1:78.1	Myb-related transcription factor, partner of profilin
Cic	1	80557664	80558744	308435	6.2E-20	chr1:78.1	capicua homolog (Drosophila)
Clip3	1	85238795	85239697	308493	6.2E-08	chr1:83.5	CAP-GLY domain containing linker protein 3
Scaf1	1	95488479	95489374	56081	2.8E-11	chr1:92.8	SR-related CTD-associated factor 1
Zc3h12d	1	250472051	250472651	308266	2.0E-06	chr1:250.4	zinc finger CCCH type containing 12D
PITX3	1	251356410	251357495	29609	4.3E-42	chr1:250.4	paired-like homeodomain 3
Nr2f2	1	125286094	125286799	113984	4.3E-10		nuclear receptor subfamily 2, group F, member 2
Pax2	1	249895584	249896463	293992	2.3E-09		paired box 2
Zfp687	2	189914894	189915882	310660	2.0E-12		zinc finger protein 687
Mov10	2	200075206	200076102	310756	1.9E-08		Moloney leukemia virus 10
Nras	2	198292829	198293429	24605	3.1E-18		neuroblastoma ras oncogene
Rai14	2	60059148	60059838	294804	8.4E-09		retinoic acid induced 14
Nfe2l2	3	58393873	58394894	83619	1.5E-10		nuclear factor, erythroid derived 2, like 2
Nr4a2	3	38872480	38873296	54278	3.1E-08		nuclear receptor subfamily 4, group A, member 2
Scrt2	3	142434466	142435066	366229	1.1E-07		scratch homolog 2, zinc finger protein (Drosophila)
Sp5	3	52695364	52696343	296510	1.2E-09		Sp5 transcription factor
Abtb1	4	122936490	122937308	297432	4.9E-09		ankyrin repeat and BTB (POZ) domain containing 1
Ing3	4	48255109	48255809	312154	2.0E-19		inhibitor of growth family, member 3
Ahdc1	5	152101596	152102486	362617	9.3E-15	chr5:151.9	AT hook, DNA binding motif, containing 1
Fbxo2	5	165237874	165238963	85273	4.9E-19		F-box protein 2
Med8	5	138890220	138890995	362575	1.3E-09		mediator complex subunit 8
NR4A3	5	64719277	64719987	58853	5.2E-16		nuclear receptor subfamily 4, group A, member 3
RGD1563216	6	108814526	108815606	500694	1.1E-45		similar to HESB like domain containing 1
Sp8	6	146168329	146168929	299499	2.1E-08		Sp8 transcription factor
Dnajc14	7	2104216	2104816	114481	9.4E-11	chr7:1.25	DnaJ (Hsp40) homolog, subfamily C, member 14
Scx	7	114503595	114504375	680712	8.6E-20	chr7:114	scleraxis
Dnajc22	7	137872861	137874254	362998	1.1E-13	chr7:136.9	DnaJ (Hsp40) homolog, subfamily C, member 22
Zfp385a	7	142249435	142250435	685474	4.4E-12	chr7:141.3	zinc finger protein 385A
Nr4a1	7	140012571	140013171	79240	1.6E-18		nuclear receptor subfamily 4, group A, member 1
Arid3b	8	61607737	61608550	367092	5.6E-09		AT rich interactive domain 3B (Bright like)
Bcl9l	8	47449901	47450705	300673	5.0E-14		B-cell CLL/lymphoma 9-like
Klhdc3	9	10051392	10053207	363192	5.2E-11	chr9:9.55	kelch domain containing 3
Foxp4	9	8507797	8508795	363185	1.2E-09		forkhead box P4
HOXB7	10	85016559	85017435	497985	4.9E-07	chr10:84	homeo box B7
Nfe2l1	10	85586351	85587341	360610	3.5E-12	chr10:84	nuclear factor, erythroid derived 2,-like 1
Scand3	12	27861394	27861994	288622	1.8E-09	chr12:27.2	SCAN domain containing 3
Higd2al1	12	1366187	1366787	688606	3.9E-10		HIG1 hypoxia inducible domain family, member 2A-like 1
Ewsr1	14	85758629	85759334	289752	1.7E-09		Ewing sarcoma breakpoint region 1
Ndrg2	15	27346566	27347666	171114	4.8E-28		N-myc downstream regulated gene 2
Zic5	15	107598237	107599053	361095	2.9E-10		Zic family member 5 (odd-paired

							homolog, Drosophila)
Id4	17	22528169	22528964	291023	4.3E-13		inhibitor of DNA binding 4
Arid4b	17	59458282	59459273	84481	9.8E-09		AT rich interactive domain 4B (Rbp1 like)
Gata6	18	2502507	2503598	29300	2.8E-09		GATA binding protein 6
LOC685382	18	15772845	15773445	685382	8.9E-13		similar to Proline-rich nuclear receptor coactivator 1
Znf521	18	5147539	5148225	307579	3.5E-08		zinc finger protein 521
LYL1	19	25113855	25114855	304663	7.9E-22	chr19:24.1	lymphoblastic leukemia derived sequence 1
Nudt7	19	41441292	41442411	361413	3.4E-15		nudix (nucleoside diphosphate linked moiety X)-type motif 7
Armcx2	X	122265349	122266438	367903	4.0E-29		armadillo repeat containing, X-linked 2
Zmym3	X	89491287	89491987	317260	3.0E-09		zinc finger, MYM-type 3
**Translation & Protein Modification**							
Tarsl2	1	120049966	120051055	308701	4.0E-13		threonyl-tRNA synthetase-like 2
Ptrh1	3	11749922	11750919	362113	7.0E-11		peptidyl-tRNA hydrolase 1 homolog (S. cerevisiae)
Rps8	5	137468053	137469043	65136	2.4E-08		ribosomal protein S8
Padi2	5	159761520	159762334	29511	6.7E-12		peptidyl arginine deiminase, type II
Rpl8	7	114953948	114955044	26962	1.0E-10	chr7:114	ribosomal protein L8
Syncrip	8	93822110	93822710	363113	9.8E-12		synaptotagmin binding, cytoplasmic RNA interacting protein
Igf2bp1	10	84714285	84715211	303477	1.6E-12	chr10:84	insulin-like growth factor 2 mRNA binding protein 1
Npm1	10	18062093	18062873	25498	2.0E-19		nucleophosmin (nucleolar phosphoprotein B23, numatrin)
Eif4g1	11	82470316	82471209	287986	1.6E-12	chr11:81.5	eukaryotic translation initiation factor 4 gamma, 1
Hnrpdl	14	10859038	10860028	305179	1.6E-18		heterogeneous nuclear ribonucleoprotein D-like
Aars	19	38413210	38414035	292023	1.1E-07		alanyl-tRNA synthetase
Mrps18b	20	2984086	2984883	294230	2.8E-26	chr20:2.4	mitochondrial ribosomal protein S18B
Rpl36	Un	25163917	25164597	58927	4.1E-24		ribosomal protein L36
**Miscellaneous & Unknown**							
RGD1565346	1	93660805	93661590	499139	1.0E-10	chr1:92.8	similar to expressed sequence C80587
RGD1565305	1	93866197	93866982	499141	9.8E-10	chr1:92.8	similar to RIKEN cDNA 4931406B18
Tmem160	1	76716621	76717399	292654	1.3E-07		transmembrane protein 160
Trim3	1	163361976	163362576	83616	5.1E-08		tripartite motif-containing 3
Tsku	1	155621658	155622558	308843	6.0E-36		tsukushin
NSCAN pred chr1.1565.a	1	176442929	176443636		1.1E-07		
RGD1307615	3	3780798	3781496	362084	9.2E-11		similar to hypothetical protein FLJ13045
RGD1311517	5	172951361	172952061	313775	1.4E-07	chr5:172	similar to RIKEN cDNA 9430015G10
Wdr8	5	171103703	171104382	366515	5.4E-08		WD repeat domain 8
NSCAN pred chr5.714.a	5	125316795	125317615		1.6E-07		
Birc6	6	20847680	20848580	313876	3.5E-13		baculoviral IAP repeat-containing 6
Crip	6	138159304	138160009	691657	2.4E-10		cysteine-rich intestinal protein
Wdr35l	6	32462311	32462988	503018	5.7E-10		WD repeat domain 35-like
NSCAN pred chr6.1025.a	6	143691197	143692302		5.8E-12		
NSCAN pred chr8.1119.a	8	123867973	123869170		3.3E-11		
NSCAN pred chr8.784.a	8	85203182	85203992		2.3E-07		
NSCAN pred chr8.1047.a	8	114860374	114861274		6.7E-13	chr8:114.3	
Nme3	10	14145576	14146376	85269	1.5E-09	chr10:14.1	non-metastatic cells 3, protein expressed in
Tmem95	10	56720709	56721596	691982	3.7E-09	chr10:55.2	transmembrane protein 95
Fam117a	10	84021838	84023150	497983	8.9E-09		family with sequence similarity 117, member A

RGD1565744	10	14488661	14489341	360494	9.5E-11		similar to RIKEN cDNA 0610007P22
RGD1311899	12	42954418	42955018	288704	2.7E-09	chr12:42.2	similar to RIKEN cDNA 2210016L21 gene
RGD1560846	12	6095843	6096542	498133	3.5E-13		similar to hypothetical protein MGC40178
rCG21620-like	12	34588608	34589612	100359816	3.0E-12		rCG21620-like
NSCAN pred chr12.007.a	12	1019708	1020388		5.4E-10		
Fam72a	13	44451758	44452849	681249	1.1E-10		family with sequence similarity 72, member A
RGD1307161	13	99339499	99340519	305031	2.0E-22		similar to 0610010K06Rik protein
NSCAN pred chr14.352.a	14	49589568	49590168		2.2E-11		
NSCAN pred chr17.082.a	17	12540665	12541742		7.5E-11		
RGD1562080	18	12945779	12946669	498827	7.3E-11		similar to Hypothetical protein CBG10141
NSCAN pred chr19.598.a	19	56135908	56136508		4.5E-08		
NSCAN chr20.066.a	20	3442365	3443350		8.6E-23	chr20:2.4	
RGD1560927	X	7709114	7709808	501507	6.1E-09		RGD1560927
NSCAN pred chrX.046.a	X	7961753	7962453		3.4E-09		
NSCAN pred chrX.568.a	X	90946064	90947069		1.6E-09		
**EST's**							
RGD1561205	5	149686351	149687140	500557	1.1E-07		
RGD1562342	7	2104216	2104816	500762	9.4E-11		similar to RIKEN cDNA 1110012D08
RGD1563441	10	56132674	56133469	497935	3.0E-07	chr10:55.2	similar to RIKEN cDNA A030009H04
TL0ABA42YC 21 mRNA sequence	20	6067533	6068428		7.7E-12		

*- DMR cluster location code is "chromosome number:cluster start in Mbp", and 2-5 Mbp in size.

Supplemental Table S2B.

List of rat sperm differential methylation regions (DMR) found in F3-generation after exposure of F0-generation to Plastics

Gene Symbol	Chr	Start	Stop	Gene ID	min p-value	DMR Cluster Location*	GeneTitle
**Apoptosis**							
Pdcd11	1	252412922	252413522	309458	1.2E-13		programmed cell death 11
Tnfrsf12a	10	12941335	12942463	302965	3.5E-12		tumor necrosis factor receptor superfamily, member 12a
Higd2a	17	16084041	16084831	290999	6.2E-13		HIG1 hypoxia inducible domain family, member 2A
**Cell Cycle**							
Cep55	1	242381886	242382486	294074	1.6E-15		centrosomal protein 55kDa
Orc4l	3	29851658	29852473	295596	1.8E-10		origin recognition complex, subunit 4-like (yeast)
Amn1	4	186612094	186612807	302032	7.8E-10		antagonist of mitotic exit network 1 homolog (S. cerevisiae)
Dock6	8	20894272	20895072	367039	9.2E-11	chr8:20	dedicator of cytokinesis 6
Cables1	18	3424659	3425645	307585	8.2E-22		Cdk5 and Abl enzyme substrate 1
**Cytoskeleton-ECM**							
Ldb1	1	251260561	251261161	309447	2.1E-22	chr1:250.4	LIM domain binding 1
Actn3	1	207491938	207492749	171009	1.9E-09		actinin alpha 3
Actl6a	2	118919059	118919939	361925	1.3E-11		actin-like 6A

Tspan33	4	56583349	56583949	500065	1.5E-14	chr4:56.25	tetraspanin 33
Actg2	4	117748353	117749060	25365	3.8E-11	chr4:116.7	actin, gamma 2, smooth muscle, enteric
Tpm2	5	60003516	60004592	500450	2.6E-07		tropomyosin 2, beta
LOC1003633 66	8	30972488	30973193	100363366	3.9E-10		amyloid beta (A4) precursor-like protein 2-like
Tpm1	8	71356826	71357518	24851	1.4E-13		tropomyosin 1, alpha
Ncam2	11	20423008	20423897	288280	3.8E-09		neural cell adhesion molecule 2
Mcoln1	12	2637728	2638418	288371	1.2E-15		mucolipin 1
Fat1	16	50588806	50589703	83720	3.1E-08		FAT tumor suppressor homolog 1 (Drosophila)
Spock3	16	29751447	29752047	306404	2.5E-06		sparc/osteonectin, cwcv and kazal-like domains proteoglycan
Tubb2a	17	37138997	37140410	498736	1.5E-33		tubulin, beta 2a
Vim	17	87846882	87848477	81818	3.1E-15		vimentin
Pcdhb15	18	30286538	30287543	291646	5.6E-21		protocadherin beta 15
Cdh11	19	2225447	2226332	84407	1.5E-14		cadherin 11
Cdh8	19	5685525	5686520	84408	9.2E-19		cadherin 8
Lrg1	Un/ 9	25672610	25673300	367455	7.7E-10		leucine-rich alpha-2-glycoprotein 1
Plin5	Un/ 9	25672610	25673300	501283	7.7E-10		perilipin 5
**Development**							
Dmpk	1	78450272	78451687	308405	8.8E-25	chr1:78.1	dystrophia myotonica-protein kinase
Six5	1	78450272	78451687	308406	8.8E-25		SIX homeobox 5
Sv2b	1	130887128	130887728	117556	2.3E-14		synaptic vesicle glycoprotein 2b
Usmg5	1	252412922	252413522	171069	1.2E-13		up-regulated during skeletal muscle growth 5 homolog (mouse)
Dkk2	2	229541018	229541911	295445	1.4E-17		dickkopf homolog 2 (Xenopus laevis)
Ntng1	2	205805922	205806522	295382	7.6E-11		netrin G1
Sv2c	2	26554292	26554989	29643	3.1E-08		synaptic vesicle glycoprotein 2c
Cbln4	3	162920485	162921468	228942	9.8E-12		cerebellin 4 precursor
Lhx6	3	15214211	15214897	311901	9.9E-10		LIM homeobox 6
Ntng2	3	8228607	8229412	311836	4.3E-19		netrin G2
SPATA2	3	158640784	158641479	114210	1.6E-26		spermatogenesis associated 2
SMO	4	56653034	56653704	25273	2.2E-12	chr4:56.25	smoothened homolog (Drosophila)
Dfnb31	5	80377382	80378089	313255	1.6E-09	chr5:79.4	deafness, autosomal recessive 31
Npc2	6	108814406	108815606	286898	1.4E-36	chr6:107.8	Niemann-Pick disease, type C2
Trps1	7	87076226	87077124	299897	2.5E-12		trichorhinophalangeal syndrome I homolog (human)
Sox14	8	104731115	104731715	300954	2.1E-09		SRY (sex determining region Y)-box 14
Per1	10	55855898	55856498	287422	4.1E-07	chr10:55.2	period circadian protein homolog 1 (Drosophila)
Nlgn2	10	56678445	56679248	117096	2.9E-10	chr10:55.2	neuroligin 2
Hoxb6	10	85032294	85033304	497986	3.9E-26	chr10:84	homeo box B6
HOXB3	10	85078411	85079388	303488	1.8E-20	chr10:84	homeo box B3
Lhx4	13	70704284	70705161	360858	9.0E-12		LIM homeobox 4
Nsg1	14	77932142	77933147	25247	2.2E-10		neuron specific gene family member 1
Irx5	19	15751735	15752415	498918	2.6E-10		iroquois homeobox 5
**Electron Transport**							
Cyp26a1	1	241947986	241948586	154985	1.6E-10		cytochrome P450, family 26, subfamily a, polypeptide 1
**Epigenetics**							
Smarca2	1	230016032	230017142	361745	1.8E-16		SWI/SNF related, matrix associated, actin dependent regulator
Dnmt3a	6	26859491	26860180	444984	3.9E-13		DNA (cytosine-5-)-methyltransferase 3 alpha
H1f0	7	117001533	117002342	24437	4.0E-11	chr7:116.3	H1 histone family, member 0
Satb2	9	55824749	55825838	501145	1.4E-13		SATB homeobox 2
Tbx2	10	74084425	74085225	303398	6.4E-41		T-box 2
ASMT	12	16815952	16816847	246281	3.6E-20	chr12:15.8	acetylserotonin O-methyltransferase
Asmtl	12	16824203	16825214	288527	1.1E-10		acetylserotonin O-methyltransferase-like
Kdm2b	12	34672398	34673309	304495	1.9E-19		lysine (K)-specific demethylase 2B

Gadd45g	17	19231639	19232721	291005	7.5E-12		growth arrest and DNA-damage-inducible, gamma
**Golgi Apparatus**							
Ap2a1	1	95403348	95404143	308578	4.4E-11	chr1:92.8	adaptor-related protein complex 2, alpha 1 subunit
B4galt2	5	138346449	138347345	313536	7.2E-07		UDP-Gal:betaGlcNAc beta 1,4-galactosyltransferase, polypeptid
**Growth Factors**							
Fgf15	1	205323456	205324556	170582	2.1E-15		fibroblast growth factor 15
Gdnf	2	57403318	57404513	25453	2.5E-25		glial cell derived neurotrophic factor
Ntf3	4	162504664	162505639	81737	3.3E-13		neurotrophin 3
WNT10B	7	137545980	137547070	315294	1.7E-13	chr7:136.9	wingless-type MMTV integration site family, member 10B
Sept11	14	16548270	16549359	305227	1.7E-11		septin 11
**Immune Response**							
Siglec10	1	93784998	93785998	292844	4.5E-12	chr1:92.8	sialic acid binding Ig-like lectin 10
Tmpo	7	28112035	28112635	25359	2.3E-10		thymopoietin
Cd276	8	62361422	62362307	315716	1.8E-09		Cd276 molecule
Lkap	10	822060	822860	170946	6.4E-10		limkain b1
Fcgr2a	13	86913190	86913875	116591	4.1E-10		Fc fragment of IgG, low affinity IIa, receptor (CD32)
RT1-CE7	20	3416588	3417188	368153	3.7E-17	chr20:2.4	RT1 class I, locus CE7
C4b	20	4103885	4104485	24233	3.9E-11	chr20:3.6	complement component 4B
RT1-A2	20	5022398	5022998	24974	1.3E-13	chr20:3.6	RT1 class Ia, locus A2
**Metabolism & Transport**							
Bcat2	1	96038195	96038795	64203	6.3E-07	chr1:92.8	branched chain aminotransferase 2, mitochondrial
Scd	1	249358774	249359374	83792	2.7E-09		stearoyl-CoA desaturase (delta-9-desaturase)
Fah	1	140875869	140876469	29383	7.8E-09		fumarylacetoacetate hydrolase
Kcnn3	2	181715841	181716441	54263	4.1E-08	chr2:180.7	potassium intermediate/small conductance calcium-activated
Acoxl	3	115365654	115366254	296138	1.3E-23		acyl-Coenzyme A oxidase-like
Slc32a1	3	149342424	149343525	83612	5.4E-12		solute carrier family 32 (GABA vesicular transporter), member 1
Mat2a	4	105744842	105745527	171347	4.4E-31		methionine adenosyltransferase II, alpha
Magi2	4	11440081	11441061	113970	7.2E-11		membrane associated guanylate kinase, WW and PDZ domain
Echdc2	5	129265151	129266066	298381	5.8E-10		enoyl Coenzyme A hydratase domain containing 2
Dhrs3	5	163340874	163341672	313689	8.1E-08		dehydrogenase/reductase (SDR family) member 3
Fut9	5	40934221	40934821	84597	1.7E-11		fucosyltransferase 9 (alpha (1,3) fucosyltransferase)
Pusl1	5	172749107	172749987	362681	5.4E-10		pseudouridylate synthase-like 1
Aldh6a1	6	108515786	108516671	81708	8.2E-11	chr6:107.8	aldehyde dehydrogenase 6 family, member A1
Pygl	6	92340725	92341609	64035	7.8E-10		phosphorylase, glycogen, liver
Syt10	7	128173824	128174714	60567	2.8E-10	chr7:126.8	synaptotagmin X
Scn3b	8	43230920	43231900	245956	4.3E-08		sodium channel, voltage-gated, type III, beta
Srr	10	62234533	62235133	303306	8.1E-09		serine racemase
KCNJ2	10	100568435	100569338	29712	8.0E-10		potassium inwardly-rectifying channel, subfamily J, member 2
Nmnat2	13	67969800	67970695	289095	2.5E-11		nicotinamide nucleotide adenylyltransferase 2
Tomm40b	13	87111787	87112679	304971	1.0E-11		translocase of outer mitochondrial membrane 40 homolog B
Enoph1	14	10859038	10859932	305177	6.9E-41		enolase-phosphatase 1
Dpysl2	15	46412626	46413517	25416	1.9E-09		dihydropyrimidinase-like 2
Kcnn2	18	39559856	39560646	54262	5.7E-10		potassium intermediate/small conductance calcium-activated

Best2	19	24802086	24803166	364973	4.5E-09	chr19:24.1	bestrophin 2
Prps2	X	47274725	47275523	24689	1.8E-06		phosphoribosyl pyrophosphate synthetase 2
**Proteolysis**							
Ube2h	4	57216643	57217348	296956	1.2E-06	chr4:56.25	ubiquitin-conjugating enzyme E2H
Metap2	7	31001102	31001702	64370	8.2E-11		methionyl aminopeptidase 2
Prssl1	7	11424819	11425640	408241	6.1E-18		protease, serine-like 1
AMZ2	10	98899341	98900231	360650	2.2E-09		archaelysin family metallopeptidase 2
Mmp17	12	28171455	28172150	288626	1.2E-10	chr12:27.2	matrix metallopeptidase 17
RGD1560350	20	17488019	17488699	365554	1.2E-09		similar to proteasome subunit iota
Pi16	20	7639255	7640255	294312	2.5E-14		peptidase inhibitor 16
**Receptors & Binding Proteins**							
Abcc6	1	96527328	96528133	81642	4.5E-09	chr1:92.8	ATP-binding cassette, sub-family C (CFTR/MRP), member 6
Esrra	1	209597756	209598356	293701	1.2E-10		estrogen related receptor, alpha
Pepd	1	87420067	87420667	292808	2.8E-09		peptidase D
Ranbp3l	2	58725173	58726193	294789	4.8E-11		RAN binding protein 3-like
Olr414	3	163798054	163799129	56821	9.3E-16		olfactory receptor 414
Spsb2	4	160931316	160932317	297592	1.0E-12	chr4:160.3	splA/ryanodine receptor domain and SOCS box containing 2
Olr1070	7	8317229	8317931	366816	3.1E-21		olfactory receptor 1070
Abcc4	15	103052860	103053460	170924	4.0E-14		ATP-binding cassette, sub-family C (CFTR/MRP), member 4
Adra1a	15	46173075	46173997	29412	9.8E-09		adrenergic, alpha-1A-, receptor
Olr1684	20	3497439	3498621	294151	1.1E-10		olfactory receptor 1684
**Signaling**							
Calca	1	172690139	172691119	24241	7.3E-13		calcitonin-related polypeptide alpha
Cnksr3	1	37957713	37958623	308113	4.4E-10		Cnksr family member 3
Gng8	1	77218978	77219578	245986	1.9E-11		guanine nucleotide binding protein (G protein), gamma 8
RAB13	2	182460590	182461570	81756	2.3E-43	chr2:180.7	RAB13, member RAS oncogene family
ANP32E	2	190715161	190716273	361999	1.4E-15		acidic (leucine-rich)nuclear phosphoprotein 32 family, member E
Egflam	2	56666338	56667335	365691	6.9E-18		EGF-like, fibronectin type III and laminin G domains
Eps8l3	2	203455748	203456642	295361	1.3E-10		EPS8-like 3 (Epidermal growth factor receptor kinase substrate
Notch2	2	192855641	192856541	29492	1.3E-16		Notch homolog 2 (Drosophila)
Dusp15	3	143297017	143298106	362238	5.8E-21		dual specificity phosphatase 15
Acap3	5	172749107	172749987	313772	5.4E-10	chr5:172	ArfGAP with coiled-coil, ankyrin repeat and PH domains 3
LOC683719	5	86273089	86273964	683719	5.6E-11		similar to RAS and EF hand domain containing
Itga7	7	2230364	2231254	81008	4.0E-08	chr7:1.25	integrin, alpha 7
Palm	7	11424819	11425640	170673	6.1E-18	chr7:9.6	paralemmin
Shc2	7	11584014	11584614	314612	1.2E-16	chr7:9.6	SHC (Src homology 2 domain containing) transforming protein 2
Mapk11	7	127442049	127442649	689314	4.7E-13	chr7:126.8	mitogen-activated protein kinase 11
Shank3	7	127816023	127816717	59312	5.0E-15	chr7:126.8	SH3 and multiple ankyrin repeat domains 3
Calcoco1	7	141470621	141471436	246047	3.8E-15	chr7:141.3	calcium binding and coiled coil domain 1
Grasp	7	139968279	139968967	192254	1.8E-07		GRP1 (general receptor for phosphoinositides 1)-associated
Anp32a	8	66488157	66489557	25379	3.8E-09		acidic (leucine-rich) nuclear phosphoprotein 32 family, member A
Snx33	8	60673757	60674357	315696	3.1E-24		sorting nexin 33
Hsp90ab1	9	11032495	11033610	301252	5.1E-10	chr9:9.55	heat shock protein 90kDa alpha (cytosolic), class B member 1
PLEKHH3	10	90191341	90192426	360634	1.3E-16		pleckstrin homology domain containing, family H (with MyTH4
Akap17a	12	16824203	16825214	288526	1.1E-10	chr12:15.8	A kinase (PRKA) anchor protein 17A
Sorbs3	15	50590321	50591217	282843	7.4E-09		sorbin and SH3 domain containing 3

Grk6	17	15237197	15237797	59076	3.7E-13		G protein-coupled receptor kinase 6
Morg1	19	24731570	24733149	288924	4.1E-08	chr19:24.1	mitogen-activated protein kinase organizer 1
Ppp1r10	20	2984169	2984883	65045	1.2E-29		protein phosphatase 1, regulatory subunit 10
Ppp1r10	20	2987593	2988193	65045	7.7E-09		protein phosphatase 1, regulatory subunit 10
Ptprs	Un	25300105	25300805	25529	9.8E-12		protein tyrosine phosphatase, receptor type, S
Gprasp1	X	1331319	1331919	171407	2.7E-09		G protein-coupled receptor associated sorting protein 1
**Transcription**							
PITX3	1	251356410	251357495	29609	3.5E-36	chr1:250.4	paired-like homeodomain 3
Ccdc21	5	152927566	152928385	362622	5.7E-18	chr5:151.9	coiled-coil domain containing 21
Fbxo2	5	165238253	165238963	85273	2.0E-08		F-box protein 2
Ccdc17	5	136879913	136881400	500528	3.1E-14		coiled-coil domain containing 17
NR4A3	5	64719197	64719987	58853	1.2E-13		nuclear receptor subfamily 4, group A, member 3
Tardbp	5	165710469	165711069	298648	2.6E-09		TAR DNA binding protein
Tceb1	5	1975203	1976200	64525	1.2E-10		transcription elongation factor B (SIII), polypeptide 1
RGD1563216	6	108814406	108815606	500694	1.4E-36		similar to HESB like domain containing 1
Batf	6	109793195	109793870	299206	1.6E-08		basic leucine zipper transcription factor, ATF-like
Scx	7	114502670	114504375	680712	6.7E-13	chr7:114	scleraxis
Maff	7	117329082	117330072	366960	6.1E-11	chr7:116.3	v-maf musculoaponeurotic fibrosarcoma oncogene homolog F
Bcl9l	8	47449801	47450705	300673	1.4E-14		B-cell CLL/lymphoma 9-like
Klhdc3	9	10052217	10053619	363192	1.4E-07	chr9:9.55	kelch domain containing 3
Foxp4	9	8507797	8509094	363185	7.5E-12		forkhead box P4
Ubtf	10	91376259	91377139	25574	1.8E-09		upstream binding transcription factor, RNA polymerase I
Scand3	12	27861394	27861994	288622	4.7E-09	chr12:27.2	SCAN domain containing 3
Higd2al1	12	1366187	1366787	688606	7.1E-07		HIG1 hypoxia inducible domain family, member 2A-like 1
Atf3	13	107225329	107225929	25389	1.9E-07		activating transcription factor 3
Ndrg2	15	27346660	27347666	171114	1.1E-25		N-myc downstream regulated gene 2
Zic5	15	107598237	107599148	361095	2.6E-17		Zic family member 5 (odd-paired homolog, Drosophila)
Ing1	16	82793987	82794978	306626	1.1E-07		inhibitor of growth family, member 1
Gata6	18	2502698	2503598	29300	6.2E-09		GATA binding protein 6
Klhl14	18	13281385	13282422	364823	1.2E-09		kelch-like 14 (Drosophila)
Fbxl8	19	35084702	35085522	498941	1.1E-12		F-box and leucine-rich repeat protein 8
Armcx2	X	122265349	122266237	367903	7.8E-33		armadillo repeat containing, X-linked 2
**Translation & Protein Modification**							
Cstf3	3	89919001	89919799	362178	1.3E-13		cleavage stimulation factor, 3' pre-RNA, subunit 3
Eef2	7	10021587	10022487	29565	1.0E-14	chr7:9.6	eukaryotic translation elongation factor 2
Rnasek	10	57077243	57078332	287453	1.3E-08	chr10:55.2	ribonuclease, RNase K
Igf2bp1	10	84714200	84715211	303477	8.0E-10	chr10:84	insulin-like growth factor 2 mRNA binding protein 1
Npm1	10	18062193	18062873	25498	7.1E-10		nucleophosmin (nucleolar phosphoprotein B23, numatrin)
Eif4g1	11	82470413	82471209	287986	4.8E-10	chr11:81.5	eukaryotic translation initiation factor 4 gamma, 1
Hnrpdl	14	10859038	10859932	305178	6.9E-41		heterogeneous nuclear ribonucleoprotein D-like
Mrps18b	20	2984169	2984883	294230	1.2E-29	chr20:2.4	mitochondrial ribosomal protein S18B
Mrps18b	20	2987593	2988193	294230	7.7E-09	chr20:2.4	mitochondrial ribosomal protein S18B
Rpl36	Un	25163917	25164597	58927	1.9E-14		ribosomal protein L36
**Miscellaneous & Unknown**							

Giyd2	1	185849246	185850026	293489	1.4E-12		GIY-YIG domain containing 2
Tsku	1	155621758	155622558	308843	2.8E-21		tsukushin
NSCAN chr1.2257.a	1	220861164	220861964		3.8E-08		
NSCAN pred chr1.948.a	1	101299220	101299820		4.1E-18		
RGD1308958	5	70448054	70449168	298020	7.5E-10		similar to chromosome 9 open reading frame 5
Birc6	6	20847680	20848580	313876	2.0E-09		baculoviral IAP repeat-containing 6
NSCAN pred chr6.009.a	6	1695151	1695930		8.6E-18		
NSCAN pred chr6.1025.a	6	143691197	143692192		4.8E-16		
RGD1308134	10	57077243	57078332	287452	1.3E-08	chr10:55.2	similar to RIKEN cDNA 1110020A23
Tmem119	12	43859955	43860555	304581	2.8E-11	chr12:42.2	transmembrane protein 119
NSCAN pred chr12.007.a	12	1019708	1020388		1.6E-08		
RGD1307161	13	99339499	99340519	305031	1.4E-19		similar to 0610010K06Rik protein
Tmem33	14	43516113	43517008	59303	2.9E-12		transmembrane protein 33
NSCAN pred chr14.127.a	14	14702118	14702812		1.2E-08		
NSCAN pred chr18.057.a	18	6993296	6994296		5.4E-08		
NSCAN chr20.066.a	20	3442365	3443350		1.3E-33	chr20:2.4	
NSCAN pred chrX.212.a	X	28736330	28736930		1.1E-08		
**EST's**							
TL0AEA6YH06 mRNA seq	18	45009969	45010947		1.4E-06		
TL0AEA9YC13 mRNA seq	18	72291368	72291968		5.0E-07		
RGD1564058	19	24731570	24733149	288925	4.1E-08		similar to cDNA sequence BC056474

*- DMR cluster location code is "chromosome number:cluster start in Mbp", and 2-5 Mbp in size.

Supplemental Table S2C.

List of rat sperm differential methylation regions (DMR) found in F3-generation after exposure of F0-generation to Dioxin

Gene Symbol	Chr	Start	Stop	Gene ID	min p-value	DMR Cluster Location*	GeneTitle
**Cytoskeleton-ECM**							
Flg	2	186309317	186310200	24641	8.5E-15		filaggrin
**Development**							
Npc2	6	108814526	108815306	286898	3.6E-15	chr6:107.8	Niemann-Pick disease, type C2
Sema3b	8	112851422	112852727	363142	3.9E-11		sema domain, immunoglobulin domain (Ig), short basic domain
**Epigenetics**							
Jmjd8	10	15093529	15094314	360498	6.1E-07	chr10:14.1	jumonji domain containing 8
Hdac3	18	30875498	30876873	84578	1.9E-08		histone deacetylase 3
**Golgi Apparatus**							
B4galt2	5	138346044	138347049	313536	1.5E-13		UDP-Gal:betaGlcNAc beta 1,4-galactosyltransferase, polypep
**Growth Factors**							
Tgfbi	17	13934717	13935412	116487	5.9E-10		transforming growth factor, beta induced
**Hormone**							
LHB	1	95892653	95894255	25329	1.4E-07	chr1:92.8	luteinizing hormone beta
**Immune Response**							
Irgc1	1	79680291	79680891	308428	8.4E-07	chr1:78.1	immunity-related GTPase family,

							cinema 1
Siglec5	1	93734203	93734913	292843	1.4E-09	chr1:92.8	sialic acid binding Ig-like lectin 5
Fcgr2a	13	86914892	86915576	116591	3.1E-11		Fc fragment of IgG, low affinity IIa, receptor (CD32)
Cd99l2	15	5672856	5673836	171485	9.1E-09		CD99 molecule-like 2
**Metabolism & Transport**							
Syt3	1	94866199	94867099	25731	3.2E-07	chr1:92.8	synaptotagmin III
Ca2	2	88092498	88093184	54231	2.1E-24		carbonic anhydrase II
Loxl3	4	117244180	117245277	312478	4.2E-11		lysyl oxidase-like 3
Clcn2	11	82429579	82430269	29232	2.7E-08	chr11:81.5	chloride channel 2
Aldh7a1	18	52310889	52311983	291450	4.9E-17		aldehyde dehydrogenase 7 family, member A1
**Proteolysis**							
DPP3	3	138285960	138287265	114591	8.4E-13		dipeptidylpeptidase 3
Pi16	20	7642807	7643887	294312	2.9E-11		peptidase inhibitor 16
**Receptors & Binding Proteins**							
Olr60	1	160632243	160632843	405017	1.1E-10		olfactory receptor 60
Chrm3	17	71070835	71071435	24260	9.1E-22		cholinergic receptor, muscarinic 3
**Signaling**							
Ppp1r14a	1	84421173	84422179	114004	8.6E-12	chr1:83.5	protein phosphatase 1, regulatory (inhibitor) subunit 14A
Ffar2	1	85881877	85882477	292794	4.3E-14	chr1:83.5	free fatty acid receptor 2
Bcar3	2	219075668	219076268	310838	1.1E-14		breast cancer anti-estrogen resistance 3
Dok1	4	117244180	117245277	312477	4.2E-11	chr4:116.7	docking protein 1
Akap6	6	73042917	73043604	64553	9.5E-85		A kinase (PRKA) anchor protein 6
CSNK1G2	7	10588530	10589932	65278	7.4E-12	chr7:9.6	casein kinase 1, gamma 2
Shc2	7	11584014	11584614	314612	3.5E-12	chr7:9.6	SHC (Src homology 2 domain containing) transforming protein 2
Rasal3	7	12968011	12968901	314596	4.2E-11		RAS protein activator like 3
Hspd1	9	53896237	53896837	63868	4.7E-10		heat shock protein 1 (chaperonin)
Grid2ip	12	11553996	11554716	288484	2.4E-08		glutamate receptor, ionotropic, delta 2 (Grid2) interacting protein
Grk6	17	15237197	15237797	59076	1.7E-13		G protein-coupled receptor kinase 6
**Transcription**							
Fes	1	136208036	136209136	361597	4.3E-08		feline sarcoma oncogene
Nras	2	198291944	198293429	24605	9.9E-13		neuroblastoma ras oncogene
Pole3	5	79520269	79520987	298098	5.3E-24	chr5:79.4	polymerase (DNA directed), epsilon 3 (p17 subunit)
Tceb1	5	1975423	1976200	64525	7.7E-35		transcription elongation factor B (SIII), polypeptide 1
RGD1563216	6	108814526	108815306	500694	3.6E-15		similar to HESB like domain containing 1
Nol10	6	41121100	41121700	313981	3.1E-12		nucleolar protein 10
**Translation & Protein Modification**							
Rpl8	7	114953948	114955044	26962	2.3E-09	chr7:114	ribosomal protein L8
Syncrip	8	93822110	93822710	363113	1.1E-10		synaptotagmin binding, cytoplasmic RNA interacting protein
Padi4	5	159616539	159617242	29512	3.6E-16		peptidyl arginine deiminase, type IV
Rpl36	Un	25163917	25164597	58927	6.0E-20		ribosomal protein L36
Arl6ip4	12	33604987	33605992	65105	2.2E-07		ADP-ribosylation-like factor 6 interacting protein 4
Rpl35	3	18794594	18795299	296709	8.2E-50		ribosomal protein L35
**Miscellaneous & Unknown**							
RGD1307797	1	85716944	85717628	361547	8.9E-08	chr1:83.5	LOC361547
RGD1560846	12	6095843	6096542	498133	1.5E-11		similar to hypothetical protein MGC40178
NSCAN pred chr14.352.a	14	49589568	49590168		4.2E-12		
NSCAN pred chr14.357.a	14	49806934	49807635		3.5E-10		
Fam129c	16	18796064	18796973	498604	8.8E-09	chr16:17.8	family with sequence similarity 129, member C

NSCAN pred chr17.082.a	17	12540665	12541742		3.6E-13		
*- DMR cluster location code is "chromosome number:cluster start in Mbp", and 2-5 Mbp in size.							

Supplemental Table S2D.

List of rat sperm differential methylation regions (DMR) found in F3-generation after exposure of F0-generation to Hydrocarbons (Jet fuel)

Gene Symbol	Chr	Start	Stop	Gene ID	min p-value	DMR Cluster Location*	GeneTitle
**Apoptosis**							
Atg9a	9	74472295	74473090	363254	1.5E-10		ATG9 autophagy related 9 homolog A (S. cerevisiae)
Unc5b	20	28130915	28131715	60630	1.1E-06		unc-5 homolog B (C. elegans)
**Cytoskeleton-ECM**							
Tspan33	4	56583349	56583949	500065	9.9E-31	chr4:56.25	tetraspanin 33
**Development**							
Sh3pxd2a	1	252646829	252647729	309460	9.3E-11		SH3 and PX domains 2A
Svs3	3	155229933	155230533	192239	3.4E-09	chr3:154.2	seminal vesicle secretion 3
Sema3b	8	112852022	112852622	363142	7.0E-10		sema domain, immunoglobulin domain (Ig), short basic domain
**Epigenetics**							
Hist1h2bn	17	50352153	50352753	291157	9.6E-18		histone cluster 1, H2bn
**Immune Response**							
RT1-O2-ps	20	2776113	2776813	414983	3.1E-22	chr20:2.4	RT1 class I, locus O2, pseudogene
RT1-CE2	20	3580516	3581116	414779	4.E-122		RT1 class I, locus CE2
**Metabolism & Transport**							
Kcnk15	3	154770968	154771568	156873	2.0E-10	chr3:154.2	potassium channel, subfamily K, member 15
Dnal1	6	108168967	108169567	685664	2.5E-08	chr6:107.8	dynein, axonemal, light chain 1
Aldh7a1	18	52311273	52311983	291450	2.5E-08		aldehyde dehydrogenase 7 family, member A1
Lcat	19	35787076	35787761	24530	1.4E-16		lecithin cholesterol acyltransferase
Gstt2	20	13218263	13218863	29487	1.7E-09		glutathione S-transferase, theta 2
**Receptors & Binding Proteins**							
Efcab4a	1	201644972	201645872	309112	1.2E-06		EF-hand calcium binding domain 4A
Olr1404	10	36543212	36544396	405065	5.4E-11		olfactory receptor 1404
Vom2r69	14	740492	741794	289433	2.4E-16		vomeronasal 2 receptor, 69
**Signaling**							
Asip	3	145443642	145444242	24152	7.2E-17		agouti signaling protein
Dab2ip	3	14667015	14668012	192126	1.8E-10		DAB2 interacting protein
Akap6	6	73042917	73043697	64553	2.E-100		A kinase (PRKA) anchor protein 6
Rhoq	6	10413845	10414445	85428	2.6E-11		ras homolog gene family, member Q
Hspa8	8	43783068	43783668	24468	2.3E-09		heat shock protein A8
Sgsm1	12	44329558	44330263	288743	1.2E-16		small G protein signaling modulator 1
Rap2a	15	105569716	105570316	114560	8.0E-16		RAS related protein 2a
**Transcription**							
Gins1	3	141545679	141546279	499914	1.0E-08		GINS complex subunit 1 (Psf1 homolog)
Klhl17	5	173066362	173067163	246757	4.2E-20	chr5:172	kelch-like 17 (Drosophila)
Foxp4	9	8573569	8574670	363185	8.4E-07		forkhead box P4
Gbas	12	27924888	27925608	498174	1.3E-10	chr12:27.2	glioblastoma amplified sequence
**Miscellaneous & Unknown**							
Tmem299b	6	101643488	101644503	503035	4.1E-21		transmembrane protein 189
LOC689986	14	2831796	2832396	689986	3.3E-06		hypothetical protein LOC689986
Tmem188	19	20262077	20262677	291914	9.1E-16		transmembrane protein 299b
LOC679647	19	14597287	14598388	679647	4.8E-18		hypothetical protein LOC679647
RGD1311257	20	11500513	11501113	294333	1.8E-09		similar to C21orf70 protein
*- DMR cluster location code is "chromosome number:cluster start in Mbp", and 2-5 Mbp in size.							
