# Supplementary material for: Transgenerational Actions of Environmental Compounds on Reproductive Disease and Identification of Epigenetic Biomarkers of Ancestral Exposures
Source: PLoS One. 2012 Feb 28;7(2):e31901. doi: 10.1371/journal.pone.0031901 (PMC3289630; doi:10.1371/journal.pone.0031901)
Supplement: Table S3 — Pathways influenced by genes associated with DMR. (PDF) [file pone.0031901.s009.pdf]

Table S3. Pathways influenced by genes associated with DMR

	All Exposures	Pesticides	Plastics	Dioxin	Hydrocarbons
**Number of unique genes in the list**	**479**	**351**	**191**	**47**	**33**
**Pathway Name**	**Number of affected genes in pathway**				
Pathways in cancer	11	8	5	2	
MAPK signaling pathway	10	8	4	1	1
Cell adhesion molecules (CAMs)	9	7	5		1
Phagosome	9	7	4	1	1
Axon guidance	9	5	2	3	2
Endocytosis	9	4	5	1	2
Focal adhesion	8	8	3	1	
Regulation of actin cytoskeleton	8	6	3	2	
Neurotrophin signaling pathway	7	7	3	2	
Olfactory transduction	7	4	3	1	1
Antigen processing and presentation	7	3	3		2
Chemokine signaling pathway	6	5	3	3	
Protein processing in endoplasmic reticulum	6	4	2		1
Neuroactive ligand-receptor interaction	6	3	1	2	
Hypertrophic cardiomyopathy (HCM)	5	5	3		
Dilated cardiomyopathy	5	5	3		
Alzheimer's disease	5	5			
Insulin signaling pathway	5	4	2	2	1
Cell cycle	5	4	2		
Viral myocarditis	5	3	3		1
Purine metabolism	5	3	1	1	
Type I diabetes mellitus	5	2	2	1	1

KEGG pathway enrichment for each exposure lineage regarding genes associated with differentially methylated regions (DMR) in F3 generation sperm. Top signaling and cellular pathways with 5 or greater total genes involved.
